# Mouse Y-Linked *Zfy1* and *Zfy2* Are Expressed during the Male-Specific Interphase between Meiosis I and Meiosis II and Promote the 2^nd^ Meiotic Division

**DOI:** 10.1371/journal.pgen.1004444

**Published:** 2014-06-26

**Authors:** Nadège Vernet, Shantha K. Mahadevaiah, Yasuhiro Yamauchi, Fanny Decarpentrie, Michael J. Mitchell, Monika A. Ward, Paul S. Burgoyne

**Affiliations:** 1 MRC National Institute for Medical Research, London, United Kingdom; 2 Department of functional genomics and cancer, Institut de Génétique et de Biologie Moléculaire et Cellulaire, Illkirch, France; 3 Institute for Biogenesis Research, University of Hawaii Medical School, Honolulu, Hawaii, United States of America; 4 Aix Marseille Université, GMGF, Marseille, France; 5 Inserm UMR_S 910, Marseille, France; Washington State University, United States of America

## Abstract

Mouse *Zfy1* and *Zfy2* encode zinc finger transcription factors that map to the short arm of the Y chromosome (Yp). They have previously been shown to promote meiotic quality control during pachytene (*Zfy1* and *Zfy2*) and at the first meiotic metaphase (*Zfy2*). However, from these previous studies additional roles for genes encoded on Yp during meiotic progression were inferred. In order to identify these genes and investigate their function in later stages of meiosis, we created three models with diminishing Yp and *Zfy* gene complements (but lacking the Y-long-arm). Since the Y-long-arm mediates pairing and exchange with the X via their pseudoautosomal regions (PARs) we added a minute PAR-bearing X chromosome derivative to enable formation of a sex bivalent, thus avoiding *Zfy2*-mediated meiotic metaphase I (MI) checkpoint responses to the unpaired (univalent) X chromosome. Using these models we obtained definitive evidence that genetic information on Yp promotes meiosis II, and by transgene addition identified *Zfy1* and *Zfy2* as the genes responsible. *Zfy2* was substantially more effective and proved to have a much more potent transactivation domain than *Zfy1*. We previously established that only *Zfy2* is required for the robust apoptotic elimination of MI spermatocytes in response to a univalent X; the finding that both genes potentiate meiosis II led us to ask whether there was *de novo Zfy1* and *Zfy2* transcription in the interphase between meiosis I and meiosis II, and this proved to be the case. X-encoded *Zfx* was also expressed at this stage and *Zfx* over-expression also potentiated meiosis II. An interphase between the meiotic divisions is male-specific and we previously hypothesised that this allows meiosis II critical X and Y gene reactivation following sex chromosome silencing in meiotic prophase. The interphase transcription and meiosis II function of *Zfx*, *Zfy1* and *Zfy2* validate this hypothesis.

## Author Summary

The mouse Y chromosome genes *Zfy1* and *Zfy2* were first identified in the late 1980s during the search for the gene on the Y that triggers male development; they encode proteins that regulate the expression of other genes to which they bind via a ‘zinc finger’ domain. We have now discovered that these genes play important roles during spermatogenesis. *Zfy2* proved to be essential for the efficient operation of a ‘checkpoint’ during the first meiotic division that identifies and kills cells that would otherwise produce sperm with an unbalanced chromosome set. Female meiosis, which does not have an equivalent checkpoint, generates a significant proportion of eggs with an unbalanced chromosome set. In the present study we show that *Zfy2* also has a major role in ensuring that the second meiotic division occurs, with *Zfy1* and a related gene, *Zfx*, on the X chromosome providing some support. In order to fulfil this function all three genes are expressed in the ‘interphase’ stage between the two divisions. In female meiosis there is no interphase stage between the two meiotic divisions but in this case essential functions during the divisions are supported by stored RNAs, so an interphase is not needed.

## Introduction

**Figure 1:**
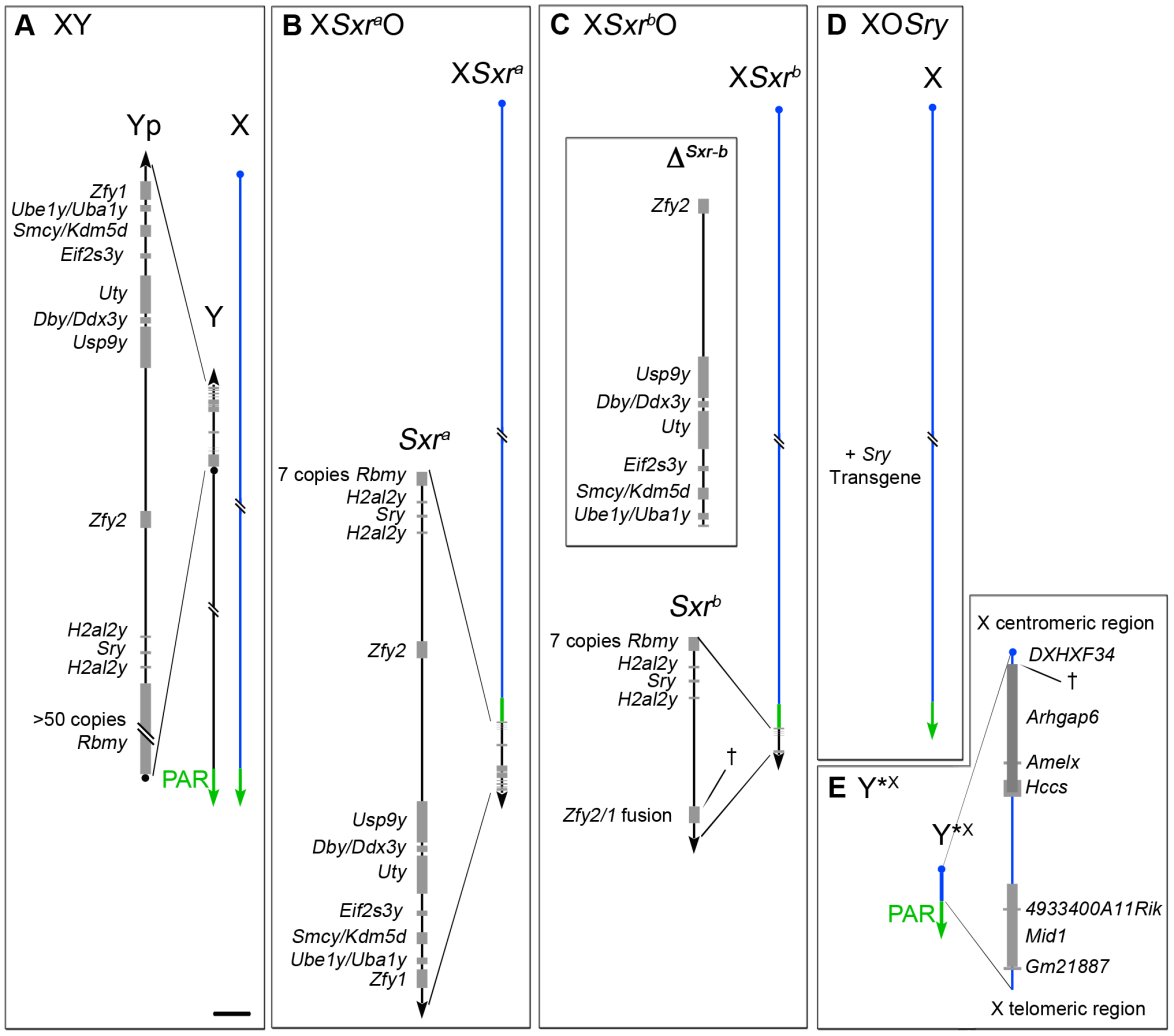
The XO and XY*^X^ mouse models. A. XY. The Y short arm (Yp) gene complement of an XY male (represented to scale in the magnified view) comprises seven single copy genes, two duplicated genes and one multi copy gene. The pseudoautosomal region (PAR) located distally on the Y long arm mediates pairing and crossing over with the X PAR during meiosis to generate the XY sex bivalent. B–D. The diminishing Yp gene complements for the three XO male mouse models that lack the Y long arm. B. X*Sxr^a^*O. The Yp-derived *Sxr^a^* sex-reversal factor, attached distal to the X PAR provides an almost complete Yp gene complement. C. X*Sxr^b^*O. The *Sxr^a^-*derived deletion variant *Sxr^b^* has a 1.3 Mb deletion (Δ*^Sxr-b^*) removing 6 single copy genes and creating a *Zfy2/1* fusion gene spanning the deletion breakpoint (†). D. XO*Sry*. This model has only one Y chromosome gene, namely the testis determinant *Sry* provided as an autosomally located transgene. E. Y*^X^. This mini sex-chromosome is an X chromosome with a deletion from just proximal to *Amelx* to within the DXHXF34 repeat adjacent to the X centromere. † represents the deletion breakpoint. This X chromosome derivative has a complete PAR that can pair with the PAR of X*Sxr^a^*, X*Sxr^b^* or X to form a ‘minimal sex bivalent’. Scale bar for magnified views is 150 kb.

Historically the realisation that there were spermatogenic factors on the human and mouse Y chromosomes distinct from the testis determinant came from the study of Y deletion variants [1], [2]. However, it was not until the search for the testis determinant that Y-encoded genes began to be identified; amongst these were the human and mouse Y genes encoding zinc finger transcription factors cloned in the late 1980s [3]–[5]. Subsequent progress in assigning spermatogenic gene functions to mouse Y-encoded genes was thwarted by a failure to disrupt Y gene functions using the emerging gene targeting techniques that had proved successful in disrupting X and autosomal gene functions, compounded by the paucity of genomic sequence data for the mouse Y chromosome. To circumvent these problems the Mitchell and Burgoyne labs established a collaboration with the aim of identifying mouse Y gene functions using a Y ‘transgene rescue’ strategy whereby Y genes were added to Y deletion variants with defined spermatogenic failure. In the context of Y genes mapping to the short arm (Yp), three XO male mouse models with diminishing Yp gene complements were utilised (Figure 1): X*Sxr^a^*O in which the X carries the Yp-derived sex-reversal factor Tp(Y)1Ct*^Sxr-a^* that provides an almost complete Yp gene complement [6], X*Sxr^b^*O males where the X carries an *Sxr^a^* derivative Tp(Y)1Ct*^Sxr-b^* in which a 1.3Mb deletion (Δ^Sxr-b^) has removed the majority of the Yp gene complement [6], [7], and XO*Sry* males in which the only Yp gene present is an autosomally located *Sry* transgene [8].

The latter two Yp-deficient models have a marked block in spermatogonial proliferation, and in 2001 we reported that this block could be circumvented by the addition of *Eif2s3y*; this Y-linked gene encodes a protein almost identical to that encoded by the X-linked gene *Eif2s3x* - a subunit of the essential translation initiation factor EIF2 [8]. Paradoxically, in both *Eif2s3y* rescue models the majority of spermatocytes complete meiosis I, whereas in the X*Sxr^a^*O ‘control’ there is a very efficient apoptotic elimination of spermatocytes at the first meiotic metaphase (MI) [9]–[11]; this apoptosis is assumed to be triggered by an MI spindle assembly checkpoint (SAC) response to the univalent X at MI [12]. This suggested that a Yp gene that was deleted or inactivated in *Sxr^b^* was necessary for an efficient apoptotic response to the univalent X, although a markedly reduced apoptotic response remained.

To identify the Yp gene that promoted the MI spermatocyte apoptosis, transgenes were tested by adding them to XO*Sry* males that carried an X-linked *Eif2s3y* transgene (here denoted as X*^E^*O*Sry*), but none of Yp genes completely removed by Δ^Sxr-b^ (Figure 1C) reinstated the apoptotic response. Focus then shifted onto *Zfy1* and *Zfy2* because the Δ^Sxr-b^ deletion breakpoints lie within these two genes, creating a transcribed *Zfy2/Zfy1* fusion gene with the encoded protein almost identical to that encoded by *Zfy1* [7], [13]. Introducing an X-linked *Zfy2* transgene into X*^E^*O*Sry* males reinstated the apoptotic response but addition of *Zfy1* had no discernible effect [11].

Further studies of the *Eif2s3y* rescue models X*^E^*O*Sry* and X*^E^Sxr^b^*O revealed that although most primary spermatocytes evaded the apoptotic response and completed meiosis I to form diploid secondary spermatocytes that entered interphase (“interphasic secondary spermatocytes”), very few secondary spermatocytes recondensed their chromosomes and underwent meiosis II [14]. We can envisage three factors that individually, or in combination, could be responsible for the meiosis II impairment: (1) The triggering of the MI SAC by the univalent X, (2) the reduced apoptotic response, and (3) the lack of a Yp gene or genes that promotes meiosis II. It is assumed that the apoptotic elimination is in some way a consequence of the prior triggering of the MI SAC [12], but with as yet no information on the molecular link between *Zfy2* expression and the apoptotic response, factors (1) and (2) are confounded. We therefore sought to check for a Yp gene requirement in a situation where the MI SAC and apoptotic response are circumvented. We have previously shown that the apoptotic elimination of MI spermatocytes in X*Sxr^a^*O males can largely be circumvented by adding a minute X chromosome derivative (denoted Y*^X^ for historical reasons) comprising a complete PAR, an X PAR boundary, a very short X-specific region and an X centromere [15]–[19] (Figure 1E). In the majority of MI spermatocytes this Y*^X^ mini-chromosome and X*Sxr^a^* had formed a sex bivalent (indicative of prior PAR synapsis and crossing over) and thus evaded the MI SAC/apoptotic elimination [19]. In the present study we therefore added this chromosome to the X*^E^*O*Sry*, X*^E^Sxr^b^*O and X*Sxr^a^*O models in order to assess if the near complete Yp gene complement of *Sxr^a^* promotes meiosis II more effectively than the two depleted Yp gene complements. This proved to be the case so we then proceeded to use Yp transgene addition to identify the Yp genes responsible for meiosis II completion.

## Results

### Yp-encoded genetic information promotes meiosis II

We will abbreviate the three models with Y*^X^ used in this study as XY*^X^ *Sxr^a^*, X*^E^*Y*^X^ *Sxr^b^* and X*^E^*Y*^X^ *Sry*. Their Yp gene complements are as shown in Figure 1B–D, except for the addition of the *Eif2s3y* transgene to the X of the latter two models (denoted X*^E^*).

**Figure 2:**
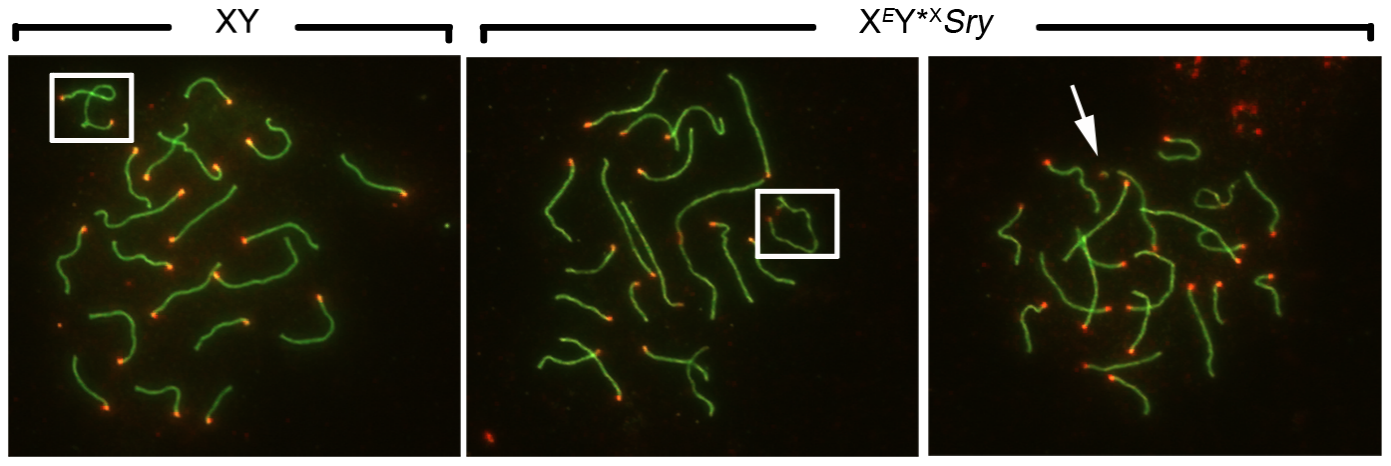
Efficiency of XY synapsis in the XY*^X^ males with varying Yp complements. Spread pachytene spermatocytes from 6 week old XY and X*^E^*Y*^X^ *Sry* testes stained with antibodies against SYCP3 (green) and CREST (red). Frames show PAR-PAR sex chromosome synapsis in XY and X*^E^*Y*^X^ *Sry* males; the arrow points to an unsynapsed Y*^X^ chromosome in an X*^E^*Y*^X^ *Sry* male.

For comparison with the published data on ploidy frequency of post-meiotic cells in the X*^E^Sxr^b^*O and X*^E^*O*Sry* models [14] we have processed the Y*^X^ complemented males at 6 weeks of age. We used a combination of centromere (CREST) and chromosome axial element (SYCP3) immunostaining, which allows PAR-PAR synapsis to be distinguished from associations between the X-derived Y*^X^ centromere and the X centromere (Figure 2). This revealed an average of 74.9% PAR-PAR synapsis with no significant difference between the three Y*^X^ complemented models and the remainder of cells either having the X and Y*^X^ PARs unpaired, or lacking an identifiable Y*^X^ (Table 1). In the latter case it is likely that the tiny Y*^X^ chromosome was lost during cell spreading.

**Table 1:** X-Y pairing efficiency in pachytene spermatocytes.

Genotype	PAR-synapsis (n)	Unsynapsed (n)	Missing Y or Y*^X^ (n)^a^	PAR-synapsis (%)^b^
XY				Average: 94.9%
1	48	2	0	96.0
2	47	3	0	94.0
3	49	1	0	98.0
4	75	1	2	96.2
5	67	3	2	93.1
6	97	5	3	92.4
X*^E^*Y*^X^ *Sry*				Average: 73.3%
1	68	29	3	68.0
2	68	27	5	68.0
3	40	5	10	72.7
4	42	13	1	75.0
5	32	5	4	78.0
6	42	8	4	77.8
X*^E^*Y*^X^ *Sxr* b				Average: 73.0%
1	72	28	0	72.0
2	28	6	6	70.0
3	82	21	4	76.6
4	27	8	1	75.0
5	73	27	2	71.6
XY*^X^ *Sxr* a				Average: 79.8%
1	81	11	2	86.2
2	74	11	6	81.3
3	49	18	2	71.0
4	71	13	4	80.7

a In a few cells, the Y or the Y*^X^ chromosome appear to be missing. Some of them might have achieved a centromere pairing with the X chromosome or have been lost during cell spreading.

b The average PAR-synapsis for all Y*^X^ bearing males (n = 15) is 74.9%.

**Figure 3:**
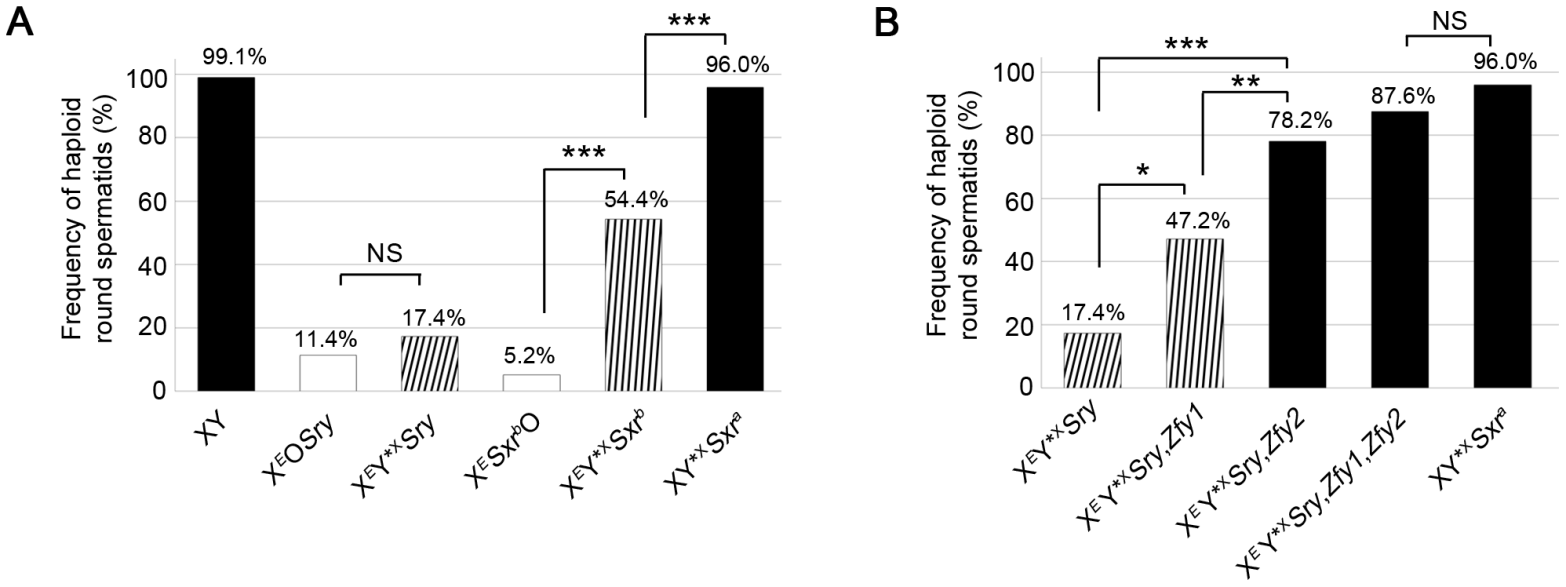
*Zfy1* and *Zfy2* promote meiosis-II in the presence of the sex chromosome pairing partner Y*^X^. Data collected after DNA quantitation of spermatids using DAPI fluorescence intensity measurement on SYCP3-labelled testis cell spreads. Pooled data expressed as percentages are shown for each genotype (n = 4). Key: in black, models with robust apoptotic elimination of MI spermatocytes with X univalents; in white, XO models with markedly reduced apoptotic response; striped, XY*^X^ models with markedly reduced apoptotic response in which the frequency was adjusted to remove spermatids derived from MI spermatocytes that had not formed an X-Y*^X^ bivalent by PAR-PAR synapsis (see Table S1). A. Percentage of haploid round spermatids found in testis of 6 week old XO and XY*^X^ males with various Yp chromosome gene contents. The data for the two XO male genotypes derive from Vernet et al., 2012 [14]. The Yp-derived *Sxr^b^* (which includes a *Zfy2/1* fusion gene encoding a ZFY1-like protein) and *Sxr^a^* (which includes *Zfy1* and *Zfy2*) promote meiosis II in the presence of Y*^X^; *Sxr^a^* is substantially more effective than *Sxr^b^*. B. Percentage of haploid round spermatids found in testis of 6 week old X*^E^*Y*^X^ *Sry* males with X-linked *Zfy* transgene additions. *Zfy1*, and to a greater extent *Zfy2*, promote meiosis II. NS, Non significant; *p≤0.05; **p≤0.01; ***p≤0.001.

To assess the efficiency of the meiotic divisions, we analyzed the ploidy of spermatids in SYCP3 and DAPI-stained spermatogenic cell spreads. The DAPI nuclear morphology of diploid spermatids is indistinguishable from that of interphasic secondary spermatocytes, but the latter have a characteristic SYCP3 staining pattern [11] and were excluded. It is important to bear in mind when assessing the consequences of the Y*^X^ additions that in an average of 25.1% of MI cells from all three models the X fails to achieve PAR synapsis with the Y*^X^ (Table 1); these cells will be subject to efficient apoptotic elimination when *Zfy2* is present, but not when *Zfy2* is absent. Our strategy was therefore to adjust the haploid frequencies of all the *Zfy2*-negative males carrying Y*^X^ by ‘removing’ the products of the 25.1% of MI cells that did not achieve PAR-PAR synapsis (see Table S1). The adjusted frequencies are presented in Figure 3; the unadjusted frequencies are available in Table S2.

Strikingly, there was no significant increase in haploid frequency in X*^E^*Y*^X^ *Sry* (17.4%) relative to X*^E^*O*Sry* (11.4%); in marked contrast the haploid frequency had significantly increased (P = 0.00059) in X*^E^*Y*^X^ *Sxr^b^* (54.4%) relative to X*^E^Sxr^b^*O (5.2%) (Figure 3A). This was an unexpected result because there was no indication from the two XO models that *Sxr^b^* potentiated meiosis II; indeed X*^E^Sxr^b^*O had a lower haploid spermatid frequency than X*^E^*O*Sry*. We conclude that meiosis II is not potentiated by the formation of a sex bivalent *per se*, but there is genetic information in *Sxr^b^* that in the context of a sex bivalent promotes the completion of meiosis II.

*Sxr^b^* has a very depleted Yp gene complement so we next wanted to assess the consequences of the Y*^X^ addition in the context of *Sxr^a^*, which provides a near complete Yp gene complement. This proved to have a much more potent effect than in the *Sxr^b^* context with the haploid frequency increasing to 96% (P = 0.00082) (Figure 3A). We conclude that there is genetic information on mouse Yp that promotes meiosis II when a sex bivalent is formed, and this is provided more effectively by *Sxr^a^* than *Sxr^b^*.

### Addition of *Zfy1* and/or *Zfy2* to X*^E^*Y*^X^ *Sry* promotes meiosis II

The protein-coding gene content of *Sxr^b^* is thought to be limited to a few copies of *Rbmy*, two copies of *H2al2y*, *Sry* and a *Zfy2/1* fusion gene spanning the *Sxr^b^* deletion breakpoint [7], [20], [21]. Because interphasic secondary spermatocytes are a very transient cell type in normal testes, there is no published information on expression of these genes at this stage. However, by RNA *in situ* analysis *Rbmy* transcripts are not detected beyond early pachytene [22] and *H2al2y* does not appear until step 6 round spermatids (Figure S1), so they are unlikely to be transcribed in interphasic secondary spermatocytes. The *Sry* transcripts present in the adult mouse testis are circular transcripts that are thought to be untranslated [23], [24]. Our initial focus was therefore on the *Zfy2/1* fusion gene, which is known to be transcribed during early prophase, is presumed to be silenced by meiotic sex chromosome inactivation (MSCI, [25]) at the beginning of pachytene, as are *Zfy1* and *Zfy2* in normal males, but is transcribed post-meiotically [7], [26]. The *Sxr^b^* deletion breakpoint is located within a 95 bp region of sequence identity between intron 5 of *Zfy2* and *Zfy1*, and the protein encoded by the fusion gene is predicted to be identical to that encoded by *Zfy1* except for the 16^th^ amino acid where a leucine replaces a phenylalanine [7], [11].

Because the *Zfy2/1* fusion gene encodes a protein nearly identical to that of *Zfy1* we first added a *Zfy1* transgene to X*^E^*Y*^X^ *Sry* to see if this mimicked the effect of *Sxr^b^* in promoting the second meiotic division in the presence of Y*^X^. This proved to be the case in that the proportion of haploid spermatids increased significantly (P = 0.01219) from 17.4% to 47.2% (Figure 3B).

Based on their DNA sequences, *Zfy1* and *Zfy2* are expected to produce transcription factors that will bind to the same target genes. We therefore also generated X*^E^*Y*^X^ *Sry* males that were transgenic for *Zfy2*, and this addition increased the haploid frequency from 17.4% to 78.2% (P = 0.00011), which is significantly higher (P = 0.00665) than that achieved with the *Zfy1* transgene (47.2%). Thus the *Zfy2* transgene promotes meiosis II more effectively than the *Zfy1* transgene. Both transgenes are single copy and inserted on the X chromosome, but we cannot assess relative transcript levels in interphasic secondary spermatocytes because of our inability to adequately purify this rare cell type. However we have previously established by qRT-PCR that the transcript level for the *Zfy1* transgene is higher than that for the *Zfy2* transgene in testes from 17.5 day-old X*^E^*O*Sry* carriers [11], so we would expect a similar excess of *Zfy1* transcripts in interphasic secondary spermatocytes. We were therefore surprised that the *Zfy2* transgene had a markedly greater effect. With the addition of both transgenes the frequency of haploid spermatids increased to 87.6% (Figure 3B; Table S2C). These results point to the combined activity of *Zfy1* and *Zfy2* as important for promoting meiosis II.

### *Zfy1*, *Zfy2* and *Zfx* are transcribed during the interphase prior to meiosis II

**Figure 4:**
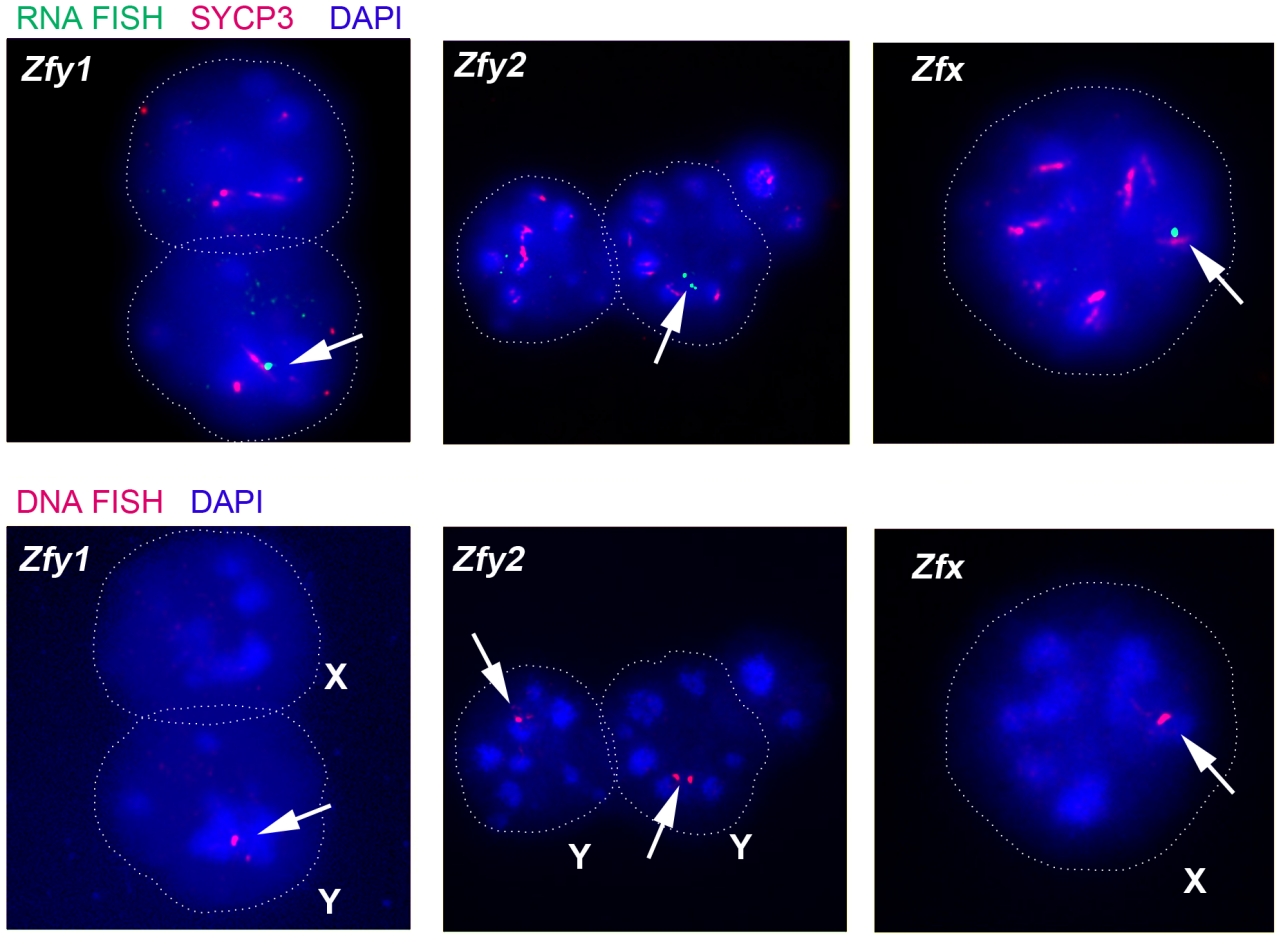
The mouse *Zfy* and *Zfx* genes are transcribed in interphasic secondary spermatocytes. Representative images of interphasic secondary spermatocyte nuclei are shown hybridized with RNA FISH probes specific for *Zfy1*, *Zfy2* or *Zfx* (arrows, top panels). Interphasic secondary spermatocytes were distinguished from diploid spermatids by staining spread spermatogenic cells from 6-week old XY males with an antibody against SYCP3 (red, top panels). The appropriate localization of the RNA FISH probe to the encoding genes was confirmed by DNA FISH (arrows, bottom panels). Nuclei are stained with DAPI (blue). X- or Y-bearing secondary spermatocytes are respectively represented by an X or a Y next to the cell.

Transcription of *Zfy1* and *Zfy2* is reportedly testis specific, at least post-natally [27]–[29]. Recently we have shown that in the adult testis this transcription is limited to germ cells, starting in leptotene spermatocytes, with more robust transcription in zygotene spermatocytes, followed by silencing in pachytene spermatocytes as a consequence MSCI; there was no resumption of transcription prior to MI, but transcription was shown to have resumed in (Y-bearing) round spermatids [7]. However, no data are available for interphasic secondary spermatocytes [14]. To assess transcription of *Zfy1* and *Zfy2* in these cells we used *Zfy1* and *Zfy2* RNA-FISH on DAPI- and SYCP3-stained testis cell spreads from XY males and confirmed the presence of the Y chromosome using *Zfy1* and *Zfy2* DNA-FISH. *Zfy1* and *Zfy2* transcription was detected in 45% and 27%, respectively, of the Y-bearing secondary spermatocytes (Figure 4; Table 2). As expected, *Zfy1* and *Zfy2* DNA-FISH signals were not observed in half of the secondary spermatocytes (X-bearing) and these also lacked *Zfy1* and *Zfy2* RNA-FISH signals. However, 92% of these X-bearing secondary spermatocytes were transcribing the related X-linked gene *Zfx* (Figure 4; Table 2).

**Table 2:** X and Y-linked ‘*Zf*’ gene expression by RNA-FISH in spread interphasic secondary spermatocytes from 6 week old XY male.

Probe	DNA FISH Negative			DNA FISH Positive		
	Total	RNA FISH Negative	RNA FISH Positive	Total	RNA FISH Negative	RNA FISH Positive
*Zfy1*	39	39	0	40	22	18 (45%)
*Zfy2*	46	46	0	45	33	12 (27%)
*Zfx*	25	25	0	26	2	24 (92%)

**Figure 5:**
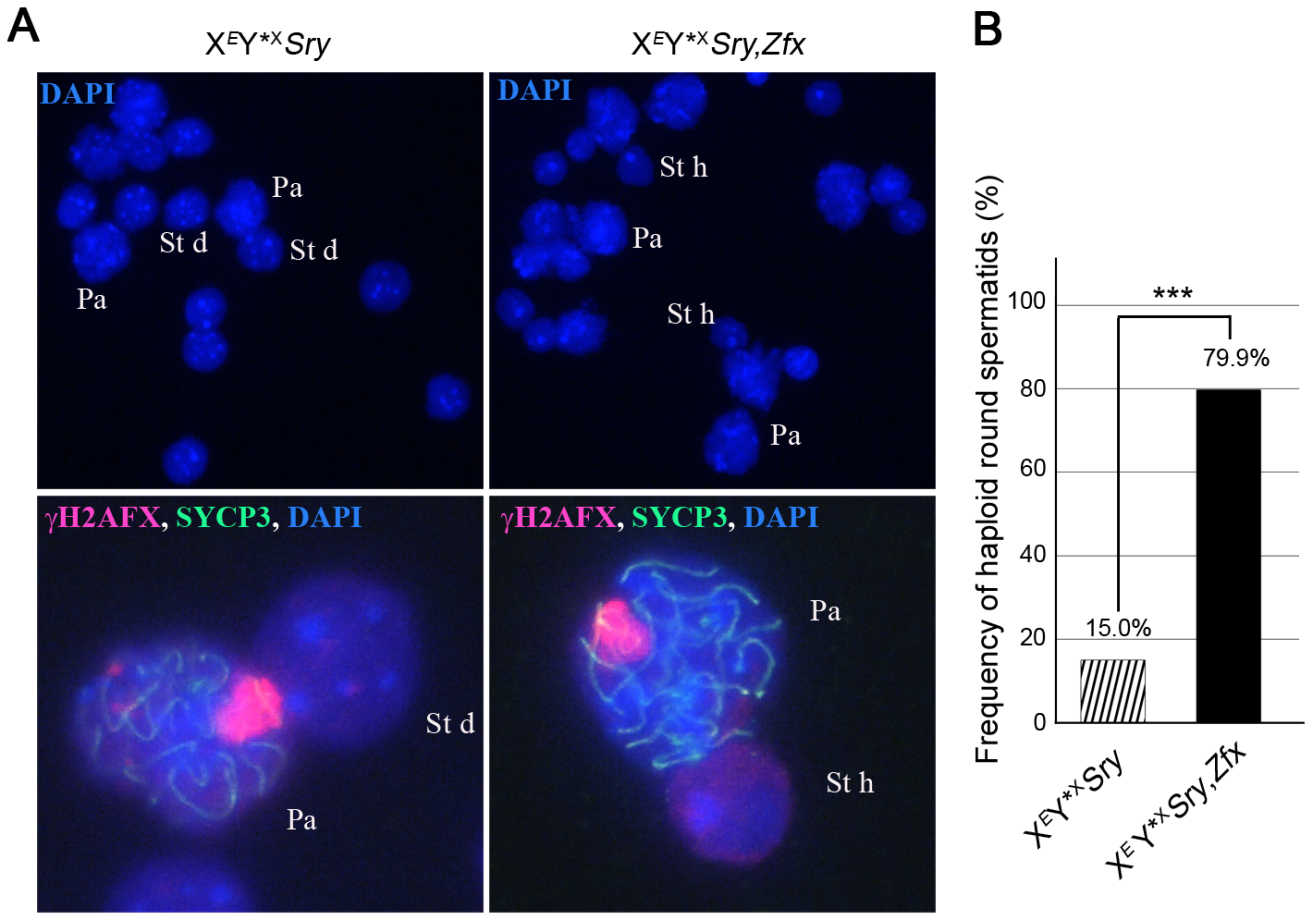
*Zfx* over-expression promotes meiosis II. A. Spread cells found in testis of 6-week old X*^E^*Y*^X^ *Sry* males without or with *Zfx* transgene. Pachytene (Pa), diploid spermatid (St d) and haploid spermatids (St h) nuclei are stained with DAPI (top panel) and higher magnifications are shown additionally labelled with γH2AFX, and SYCP3 antibodies (bottom panel). B. Percentage of haploid round spermatids found in mice from panel A (see also Table S2D). Key: in black, X*^E^*Y*^X^ *Sry*,*Zfx* transgenic males have a robust apoptotic elimination of the ∼25% of MI spermatocytes that have an X univalent (see Figure S5); striped, X*^E^*Y*^X^ *Sry* males have a markedly reduced apoptotic response so the frequency was adjusted as detailed in Table S1. The addition of the *Zfx* transgene significantly increases (***p≤0.00001) the proportion of haploid round spermatids.

Our finding that *Zfx* is also expressed in interphasic secondary spermatocytes raised the question as to whether *Zfx* also promotes the second meiotic division. We had available a *Zfx* transgenic line with 7 copies of a *Zfx* genomic BAC inserted on an autosome. As expected for an autosomally located X-chromosome-derived transgene it was exempt from MSCI, and was expressed in pachytene cells (Figure S2A,B); like the endogenous *Zfx* gene it was expressed in interphasic secondary spermatocytes (Figure S2C). We added this transgene to X*^E^*Y*^X^ *Sry* males and the proportion of haploid spermatids increased from 15.0% (in non-transgenic siblings) to 79.9%, showing that *Zfx* can promote meiosis II (Figure 5A,B; Table S2D); we therefore consider it likely that the endogenous *Zfx* also has a minor role in promoting meiosis II.

### The *Zfy2* transactivation domain is much more potent than that of *Zfy1*

**Figure 6:**
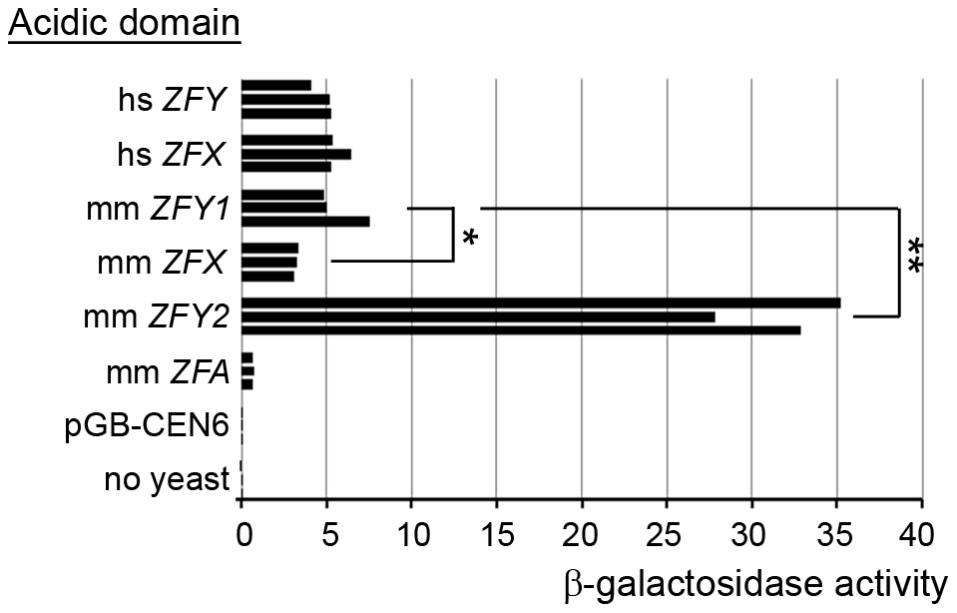
*Zfy2* acidic domain is a much more potent transactivator than other *‘Zf’* acidic domains. Levels of β-galactosidase induced by the Gal4-DNA-binding domain on its own (pGB-CEN6; negative control) or fused to an acidic domain from one of six different ZF isoforms from human (hs) or mouse (mm). Among the mouse sex-linked genes, *Zfy2* has a substantially more potent activation domain than *Zfy1*, and *Zfx* is significantly less potent than *Zfy1*. mm ZFA derives from the autosomal *Zfa* gene that originated from a retroposed X transcript. *p≤0.05; **p≤0.01.

We were struck by the much more potent effect of *Zfy2* as compared to *Zfy1* in promoting meiosis II. Based on an *in vitro* assay it was previously reported that *Zfy2* encodes a protein with a much more potent transactivation (TA) domain than that of *Zfx*, but *Zfy1* was not assayed at that time [30]. We have therefore used a similar *in vitro* assay to compare the transactivation domains of mouse *Zfx*, *Zfy1*, *Zfy2* and the autosomal *Zfa* (originating from a retroposed X transcript [31]–[35]), and have also compared these with the transactivation domains of human *ZFX* and *ZFY* (Figure 6). We confirmed the expression of all the ZF-Gal4 fusion proteins by western blot analysis (Figure S3). The assay revealed that the TA domain of mouse *Zfy1* has a similar activity to human *ZFX* and *ZFY*. Strikingly, the mouse *Zfy2* TA domain is 5.5-fold more active than that of mouse *Zfy1*, and is ∼10-fold more active than that of mouse *Zfx.* The TA domain of the putative ZFA protein proved to have a very weak TA activity. A single nucleotide deletion near the beginning of the ZFY/ZFX open reading frame of *Zfa* actually makes it very unlikely to translate a protein that includes the zinc finger DNA binding domain, which would preclude binding to target genes. *Zfa* is now flagged as a pseudogene in Genbank (accession no. NR_037920).

## Discussion

**Figure 7:**
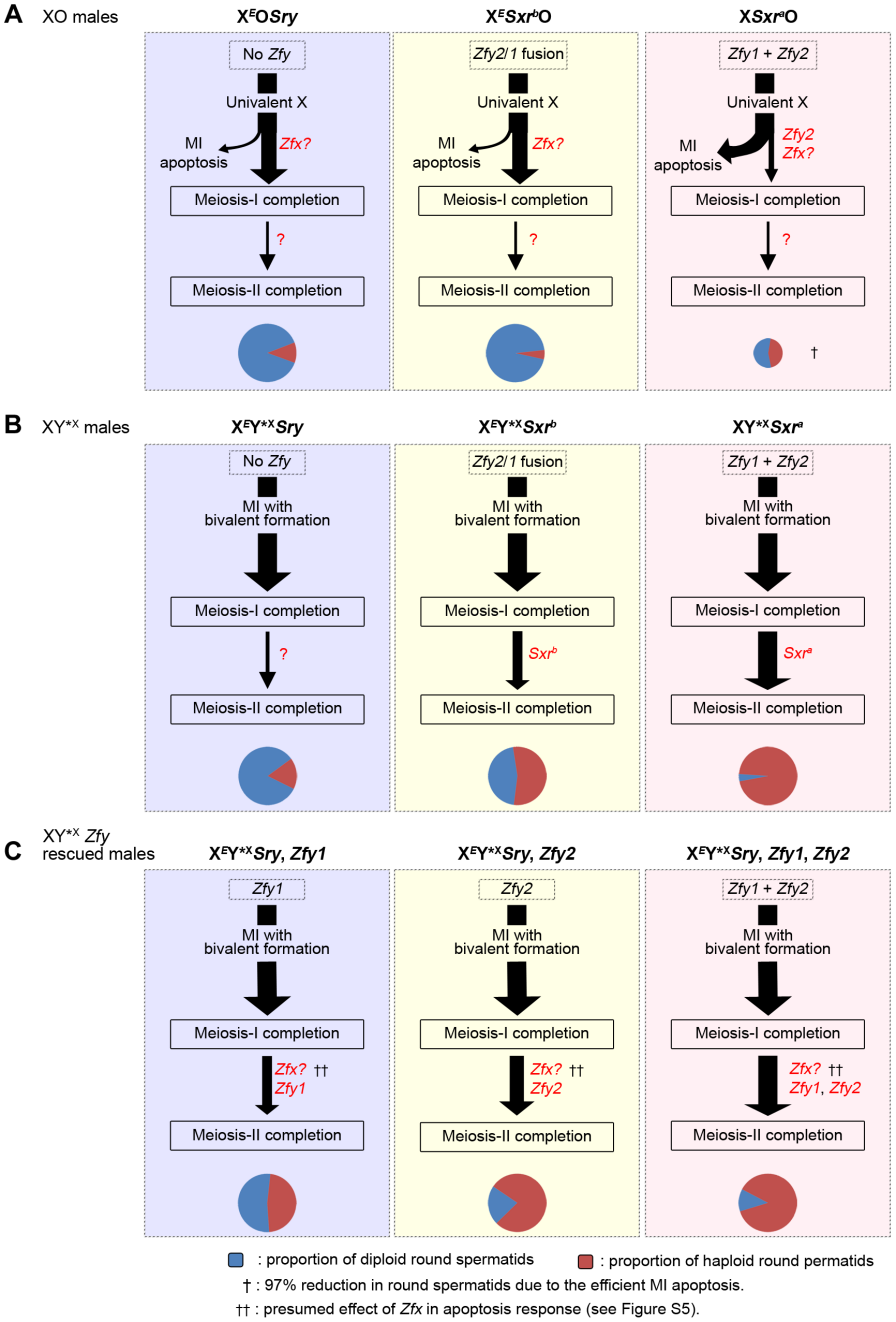
A summary of the meiotic outcome in XO and XY*^X^ males with varying Yp gene content. Throughout this figure the thickness of the arrows indicates the proportion of cells progressing from one step to the next and the cheeses at the bottom represent the proportion of haploid and diploid spermatids. Size of the cheese indicates the relative success of the different models in meiosis completion. A. XO models. In X*^E^*O*Sry* and X*^E^Sxr^b^*O males the majority of spermatocytes complete meiosis I because of the reduced apoptotic response at MI due to the absence of *Zfy2* [11]. *Zfx* expression is likely responsible for the residual apoptotic response (see Figure S5). The majority of spermatocytes then arrest at the interphase between meiosis I and meiosis II. This could be a consequence of the prior triggering of the MI SAC by the univalent X, the reduced apoptotic response due to the absence of *Zfy2*, or the lack of a Yp gene or genes that promotes meiosis II. In X*Sxr^a^*O males there is a very efficient apoptotic elimination of spermatocytes at MI so that very few complete meiosis I and this results in a 97% reduction in the number of spermatids. This precludes any firm conclusion as to a role for Yp genes for completion of meiosis II because the apoptotic elimination may have had a bias towards removing MI cells that were otherwise destined to arrest at the following interphase. B. XY*^X^ models. In these models the spermatocytes that form a sex bivalent circumvent the MI SAC/apoptotic response and complete meiosis I. This reveals that *Sxr^a^* strongly promotes meiosis II, thus confirming that a gene or genes on Yp promotes meiosis II. Surprisingly *Sxr^b^*, which did not promote meiosis II in X*^E^Sxr^b^*O males, does so now that the apoptotic response is circumvented by formation of an X-Y*^X^ sex bivalent. C. The XY*^X^ *Sry ‘Zf’* transgene addition models. These transgene additions revealed that *Zfy1* and *Zfy2* are the genes on Yp that promote meiosis II with *Zfy2* the more effective. *Sxr^b^* includes the *Zfy2/1* fusion gene that encodes a ZFY1-like protein, whereas *Sxr^a^* includes *Zfy1* and *Zfy2*, thus explaining the more potent effect of *Sxr^a^* in promoting meiosis II. *Zfx* over-expression also promotes meiosis II (Figure 5) making it likely that the endogenous *Zfx* also does so to some degree.

Our previous study of meiotic progression in the three XO male models with varying Yp gene complements revealed that the majority of spermatocytes in the Yp gene deficient models X*^E^*O*Sry* and X*^E^Sxr^b^*O reached the interphase that precedes meiosis II; however, they failed to recondense their chromosomes to enable completion of meiosis II and instead formed diploid round spermatids [14]. Formally, the failure to undergo meiosis II could be a consequence of the prior triggering of the MI SAC by the univalent X, the reduced apoptotic response due to the absence of *Zfy2*, or the lack of a Yp gene or genes that promotes meiosis II (Figure 7A). The aim of the present study was to check specifically for a Yp gene requirement by circumventing the MI SAC and apoptotic responses; for this we added a minute PAR-bearing X chromosome derivative (Y*^X^) to all three XO models to enable formation of a sex bivalent without altering the Yp gene complement. This established that there is genetic information present in *Sxr^a^* and *Sxr^b^* that promotes meiosis II, but *Sxr^a^* was more effective (Figure 7B). Yp transgene additions to X*^E^*Y*^X^ *Sry* males then identified *Zfy1* and *Zfy2* as the Yp genes responsible with *Zfy2* having the more potent effect (Figure 7C).

**Figure 8:**
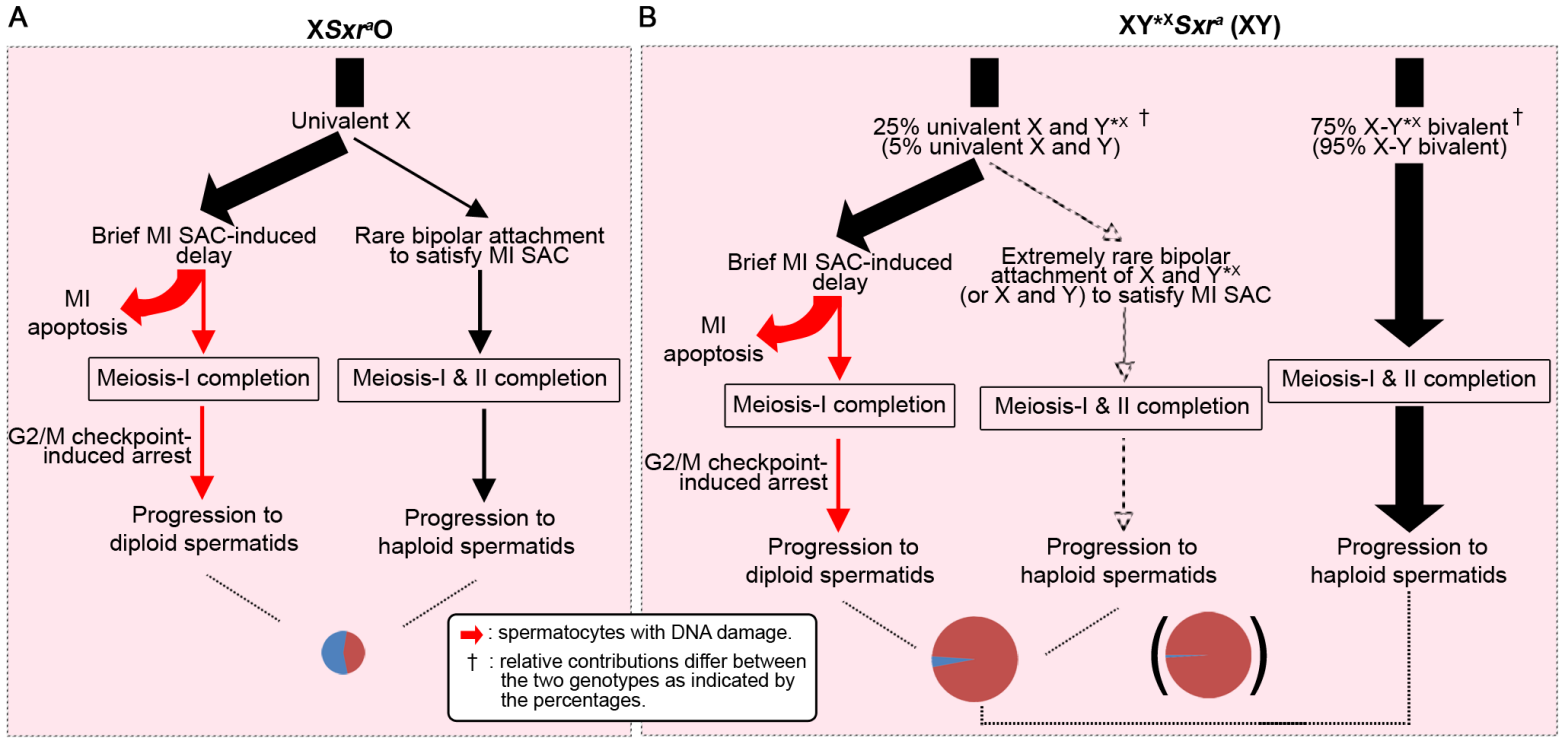
A combined MI SAC and G2/MII checkpoint model to explain the consequences of the male-specific apoptotic response to spermatocytes with univalent chromosomes at MI. To illustrate the model we consider the consequences of these two checkpoint responses in X*Sxr^a^*O, XY*^X^ *Sxr^a^* and XY males, all of which have a complete *‘Zf’* gene complement. Red arrows denote DNA damage. The cheeses at the bottom represent the proportion of haploid and diploid spermatids. **A.** In X*Sxr^a^*O males each MI spermatocyte will have a univalent X that is expected to trigger the MI SAC and cause a brief delay in MI progression. We propose that this delay is detected by the surrounding Sertoli cell, which initiates a robust *Zfy2*+*Zfx*-dependent apoptotic response. In order to explain the mix of diploid and haploid spermatids originating from the very few surviving MI spermatocytes we propose: 1) Rare MI spermatocytes complete meiosis I with apoptotic DNA damage that triggers a G2/MII checkpoint in the subsequent interphase and blocks progression to MII – these interphasic secondary spermatocytes then enter spermiogenesis to form diploid spermatids. [The number of such cells was elevated in the XO models lacking *Zfy2* in Figure 7]. 2) In rare cases some MI spermatocytes evade the MI SAC and apoptosis by achieving bipolar attachment to the spindle – these complete meiosis I and meiosis II to form haploid spermatids. **B.** In XY*^X^*Sxr^a^* males (25% of MI spermatocytes with univalent X and Y*^X^) and XY males (5% of MI spermatocytes with univalent X and Y) the MI cells with univalents will follow the pathways 1) and 2) above, although the likelihood of both univalents achieving bipolar attachment to the spindle will be much lower. The MI cells that have formed a sex bivalent will progress through both divisions to form haploid spermatids (unless they have a pair of autosomal univalents in which case pathways 1 and 2 apply).

We attribute the difference in potency between *Sxr^a^* and *Sxr^b^* to the presence of *Zfy1* and *Zfy2* in *Sxr^a^* whereas *Sxr^b^* only has the *Zfy2/Zfy1* fusion gene that encodes a protein almost identical to *Zfy1* [7], [11]). However, we were struck by the fact that *Sxr^b^* did not promote meiosis II in the absence of Y*^X^; this implies that the triggering of the MI SAC, and/or the reduced apoptotic response, impairs progression through meiosis II. In order to distinguish between these possibilities it is informative to consider what happens in XO females where there is an MI SAC response but no apoptotic response. XO female mice are fertile and produce XO (and XX) daughters, so that some XO oocytes must complete meiosis I and meiosis II. Furthermore, although some X univalents achieve bipolar attachment to the spindle (which is expected to satisfy the MI SAC), this is not a prerequisite for the completion of meiosis I [36]. This is in agreement with accumulating data for female mice showing that the MI SAC does not maintain arrest until all kinetochores have achieved appropriate attachments to the spindle - anaphase can proceed in the presence of one (or a few) univalents [37]–[41]. Thus XO oocytes can complete meiosis I to generate MII oocytes with either an XX sex chromosome complement (i.e. two X chromatids) or lacking an X chromosome, both of which should be able to complete meiosis II without triggering an MII SAC response. This suggests that in the three XO male models the triggering of the MI SAC *per se* would not impair meiosis II. We therefore favour the view that the addition of the Y*^X^ to the X*^E^Sxr^b^*O model increases the haploid spermatid frequency from 5.2% to 54.4% because the formation of an X*Sxr^b^*/Y*^X^ bivalent avoids the reduced apoptotic response. We envisage that the reduced DNA damage at MI as a consequence of the reduced apoptotic response is usually insufficient to trigger elimination at MI, but is sufficient to trigger a G2/M DNA damage checkpoint (reviewed in [42]) at the post meiosis I interphase and block progression to MII. The arrested interphase cells then enter spermiogenesis as diploid spermatids. Unfortunately we cannot use these models to assess the ultimate fate of the diploid spermatids, because in the absence of the Y long arm there is marked over-expression of X and Y genes due to the absence of the repressive effects of *Sly*, which is present in >50 copies on the Y long arm [43], [44], and this (together with Yp gene deficiency) results in severely perturbed spermiogenesis [14]. Figure 8 summarises how we see these MI and G2/MII checkpoint responses operating in males with a normal ‘*Zf*’ gene complement (X*Sxr^a^*O, XY*^X^ *Sxr^a^* and XY).

The finding that both *Zfy2* and *Zfy1* promoted meiosis II was surprising because at MI only *Zfy2* promotes the robust apoptotic elimination of spermatocytes with a univalent X chromosome [11]. What is the basis for the resurrection of *Zfy1* function in the short interval between MI and meiosis II? Our previous RNA FISH analyses of nascent nuclear transcripts on spread spermatogenic cells from normal XY males [7] have established that *Zfy1* and *Zfy2* are transcribed in all mid-late zygotene nuclei, but this ceased in pachytene nuclei – an expected consequence of meiotic sex chromosome inactivation (MSCI – reviewed by [25]), and remained undetectable right through MI. The role of *Zfy2* in the apoptotic response to univalence at MI must therefore be a consequence of transcriptional changes mediated by ZFY2 translated from these zygotene transcripts. *Zfy1* and *Zfy2* have the same predicted DNA target sequences, so if both are robustly expressed during zygotene, why is the apoptotic role limited to *Zfy2*? A plausible explanation is provided by our finding that during the pre-pachytene phase of transcription, alternative splicing of *Zfy* transcripts leads to ∼81% of *Zfy1* transcripts lacking exon 6 with the encoded protein lacking transactivation (TA) activity, whereas ∼96% of *Zfy2* transcripts have exon 6 and thus a functional TA domain [7]. Our current TA domain analysis further demonstrates that the few full length *Zfy1* transcripts that are produced during zygotene generate a protein with a much less potent TA domain than that of *Zfy2*. In view of this it is reasonable to conclude that *Zfy1* function in meiosis II is based on the *de novo* transcription in interphasic secondary spermatocytes (and that this also applies to *Zfy2*). It also implies that at this stage there is a greater preponderance of *Zfy1* transcripts with exon 6 that encodes the TA domain; this is supported by our previous finding of a 3.7-fold increase in such transcripts in pubertal testes between 20dpp and 27dpp, which covers the period when transcripts from interphasic secondary spermatocytes and round spermatids should progressively increase as a proportion of the total testis RNA [7].

Given that *Zfx*, *Zfy1* and *Zfy2*-encoded transcription factors are predicted to bind the same target sequences, it is to be expected that in tissues where they are all expressed they will regulate the transcription of the same genes. The extent to which they transactivate target genes will be dependent on the relative potency of their TA domains (*Zfx*<*Zfy1*<*Zfy2*); the protein encoded by *Zfy1* lacking exon 6 would be expected to bind but not transactivate, and could thus function as a competitive inhibitor of the three full length ZF proteins [7]. In XY males, all three sex-linked ‘*Zf*’ genes are transcribed in zygotene spermatocytes with a predominance of *Zfy1* transcripts lacking exon 6 (Figure S4 and [7]), and in interphasic secondary spermatocytes (Figure 4 and Table 2) in which full length *Zfy1* transcripts are thought to be more prevalent. Does the *Zfx* transgene addition support the expectation that *Zfx* will contribute to *‘Zf’*-mediated functions at MI (indirect) and during meiosis II (potentially direct)? We have already presented the data showing a marked promotion of meiosis II by the *Zfx* transgene (Figure 5) and there is also a marked promotion of the apoptotic response to X univalence at MI (Figure S5), which clearly demonstrates that *Zfx* is able to contribute to these functions. [The marked promotion of these functions in both cases is unsurprising given that the transgene is present in 7 copies and that its autosomal location is associated with extension of transcription through to just prior to MI, together with exemption from the MSCI-dependent repression that affects the X and Y chromatin of interphasic secondary spermatocytes (see below).] Thus it is reasonable to conclude that the endogenous *Zfx* also contributes to these functions; indeed, in the X*^E^*O*Sry* model there is some MI apoptosis [11] and in the X*^E^*Y*^X^ *Sry* model there is some progression through meiosis II (17.4% haploid spermatids, Figure 3A). A role for the endogenous *Zfx* in spermatogenesis has also been suggested based on the reduced sperm count in *Zfx* knockout males, although this effect is confounded with severe growth deficiency [45].

Although Monesi reported transcription in interphasic secondary spermatocytes in the 1960s [46], [47], other than our finding that there is de novo transcription of the multi-copy mouse Y gene *Sly* in interphasic secondary spermatocytes [48], we are not aware of any published data giving information on which genes are actively transcribed at this stage. It has previously been concluded based on Cot1 RNA FISH assessments of global transcription in interphasic secondary spermatocytes that the autosomal chromatin is actively transcribed, whereas the X and Y chromatin remains substantially repressed; the repression of the sex chromosomes has been shown to be dependent on the prior MSCI, and is carried through into round spermatids (‘post-meiotic sex chromosome repression’) [49]–[51]. This raises the possibility that the *de novo* transcription of the ‘*Zf*’ gene family represents a selective reactivation. As a first look at this issue we assessed *de novo* transcription of *Mtm1* (X-linked) and *Uty* (Y-linked) in interphasic secondary spermatocytes and round spermatids, with *Zfx* serving as a positive control. This revealed that these two genes are also transcribed in interphasic secondary spermatocytes, but it is noteworthy that for all three genes the frequency of RNA FISH positive cells was higher in interphasic secondary spermatocytes (*Zfx* 90%; *Mtm1* 67%; *Uty* 88%) than in round spermatids (*Zfx* 32%; *Mtm1* 36%; *Uty* 60%) (Table S3). These preliminary data are consistent with: (1) there being a partial relaxation of sex chromosome silencing during the late diplotene–MI period, counterbalanced by the global transcriptional repression associated with the condensation of the metaphase chromosomes, (2) the decondensation of the chromosomes in interphasic secondary spermatocytes allowing strong transcription from the autosomes, but weaker transcription from the sex chromosomes because of the MSCI carry-over effect; and (3) further repression of the sex chromosomes in round spermatids due to the repressive chromatin changes driven by the multi-copy Y gene *Sly* [44].

The marked increase in transactivation activity of *Zfy2* relative to *Zfx* and *Zfy1* (Figure 6 and [30]) raises some interesting questions in an evolutionary context. The autosomal ‘*Zf*’ precursor of *Zfy* and *Zfx* is thought to have been added to the PAR after the separation of the eutherian and marsupial lineages 193–186 million years ago, and that with further PAR additions and rearrangements it became located in the non-recombining regions of the X-Y pair [52], [53]. In eutherian mammals the X-linked genes with retained Y-linked homologues are typically exempt from X dosage compensation, suggesting a constraining dosage requirement in somatic tissues, and in most eutherian mammals this is known or is presumed to be true for *Zfx* and *Zfy* [54], [55]. However, around 40–70 million years ago in the myomorph rodent lineage, *Zfx* became subject to X-dosage compensation and the *Zfy*-encoded proteins diverged [30], [55]–[59]. Furthermore, the divergence in *Zfy* protein sequence is more marked in the highly acidic amino terminal TA domain that activates target genes, than in the carboxy terminal zinc finger domain that mediates binding to DNA. Here we have shown that in *Mus musculus* this divergence is associated with increased TA activity and that this is much more marked in *Zfy2* than in *Zfy1*. Given that in mature male mice expression of the *Zfy* genes has only been detected in testes [27]–[29], specifically in the germ-line [7], this implies that there was a strong selective force in spermatogenic cells for improved TA activity. This male germ-line specific selective force is likely to have been MSCI, which will have affected *Zfx* as well as *Zfy1* and *Zfy2*. For a zinc finger transcription factor needed for meiosis II that is dependent on transcription during the brief interphase between meiosis I and meiosis increasing the transactivation activity would be a major advantage. The TA domain of *Zfx* is likely precluded from responding to the selection because of a dosage sensitive role in somatic cells. On the other hand, the spermatogenic cell specific expression of the Y-encoded genes in the post natal testis allowed their TA domains to increase in activity, but the TA domain of *Zfy2* has responded much more than that of *Zfy1*.

Given the importance of ‘*Zf*’ gene transcription during the interphase between meiosis I and meiosis II in male meiosis, it is intriguing that female mice (and female mammals generally) have no interphase between the two meiotic divisions. We previously hypothesized that the presence of an interphase between the two meiotic divisions in male mammals would be essential if there are meiosis II critical genes on the sex chromosomes, because they would have been transcriptionally silenced (MSCI) during the preceding ∼8 days [14]. However, the two meiotic divisions in females are dependent on RNAs produced and stored during oocyte growth [60], and it may be this dependence on stored RNAs that has enabled female meiosis to dispense with the interphase.

In conclusion, our present findings provide evidence for a specific requirement for *Zfx* and *Zfy* expression in the interphase between meiosis I and meiosis II, for meiosis II to be efficiently completed. We have also provided additional evidence for a marked divergence in the functionality of the three ‘*Zf*’-encoded transcription factors, with *Zfx* providing a dosage constrained somatic role with only a minor contribution to sex-linked ‘*Zf*’ gene function in spermatogenesis, *Zfy1* developing a dual role in spermatogenesis via alternative splicing to produce activatory and repressive proteins (see also [7]), and *Zfy2* becoming a super-active transcription factor (see also [30]) to enable it to function in the face of the repressive effects of MSCI and the linked post-meiotic sex chromosome repression. There are undoubtedly further sex-linked ‘*Zf*’ gene functions to be discovered so the identification of the direct targets of the ‘*Zf*’-encoded transcription factors is a high priority. This has been thwarted by a failure to obtain specific antibodies for chromatin immunoprecipitation analyses, but transgenes encoding tagged versions of the proteins should provide a way forward. We have also presented a case for their being a G2/M DNA damage checkpoint operating in the interphase between meiosis I and meiosis II that prevents progression to MII if there is unrepaired DNA damage present; our XO mouse models together with the recent first report of a successful method for the targeted disruption of a single copy Y gene [61], will be invaluable for investigating this further. Some intriguing recent data obtained with the X*^E^Sxr^b^*O model suggested that following the injection of diploid spermatids (almost certainly together with interphasic secondary spermatocytes) into eggs (“ROSI”), a proportion of the cells completed the second meiotic division in the egg, thus avoiding triploidy which is lethal in early pregnancy [62]. The egg provides the cellular machinery for DNA damage repair by non-homologous end joining (NHEJ) [63], which would be expected to release the proposed G2/M DNA damage checkpoint arrest in the X*^E^Sxrb*O model. However, this pathway of repair is inherently mutagenic [64], which may have important ramifications for the use of ROSI with cells harboring such DNA damage if they were unintentionally used when treating human male infertility.

## Materials and Methods

### Ethics statement

All animal procedures were in accordance with the United Kingdom Animal Scientific Procedures Act 1986 and were subject to local ethical review.

### Mice

Aside from the mice with *Sxr^a^* or *Sxr^b^* attached to the Y*^X^ chromosome (see section (1) below), the mice in this study have an outbred MF1 (NIMR colony) background. The XY*^X^ males with varying Yp gene complements (Figure 1) were produced by either 1 or 2 below, and the *Zfy* and *Zfx* transgene additions to X*^E^*Y*^X^ *Sry* males are described in 3.

(i) Mating XX females to ‘X*Sxr^a^*Y*’ males, or (ii) Mating XX females homozygous for the X-linked *Eif2s3y* transgene to ‘X*Sxr^b^*Y*’ males. The fathers used in these crosses are unique genotypes generated specifically for this study to enable a more efficient production of males carrying the X chromosome derivative (Y*^X^, see below) and the Yp-derived sex reversal (*Sxr*) factors. These unique genotypes are males [15], [16], [18] with either the Tp(Y)1Ct*^Sxr-a^* sex-reversal factor [65], or the Tp(Y)1Ct*^Sxr-b^* sex-reversal factor [6], [13], attached distal to the X PAR (denoted X*Sxr^a^*Y* and X*Sxr^b^*Y* respectively). X*Sxr*Y* males have a Y chromosome ‘hijacked’ by an X centromere attached distal to a rearranged PAR. One of the recombinant sex chromosomes generated is the minute ‘Y*^X^’ chromosome comprising a complete PAR with an X PAR boundary, a very limited amount of X-specific DNA, and an X centromere (Figure 1E). In these X*Sxr*Y* males the recombination event generating the Y*^X^ adds the *Sxr^a^* or *Sxr^b^* factor distal to the Y*^X^ PAR. In producing the X*Sxr*Y* fathers the *Sxr* factors were passed through females heterozygous for the X-autosome translocation T(X;16)16H as previously described [66], [67], and this introduced some ‘non-MF1’ genetic background. The resulting X*^E^*Y*^X^ *Sxr^b^* and XY*^X^ *Sxr^a^* offspring were 87.5% MF1. A detailed description of the production and characteristics of these X*Sxr^a^*Y* and X*Sxr^b^*Y* males is in preparation.
Mating XY*^X^ females [15], [16], [18] carrying an X-linked GFP transgene marker [68] to: (i) ‘X*^E^*Y^Δ*Sry*^ *Sry*’ males that have the X carrying an *Eif2s3y* Y-genomic BAC transgene [8], a Y-chromosome with an 11 kb deletion removing *Sry* (*dl1Rlb*) [69], [70], and an autosomally located *Sry* transgene [Tg(Sry)2Ei] [71]; this cross produces X*^E^*O*Sry* and X*^E^*Y*^X^ *Sry* males that do not exhibit GFP florescence when examined using GFP goggles (FHS/EF-2G2: Biological Equipment Maintenance and Service Ltd, Budapest, Hungary). (ii) ‘X*^E^*Y*Sxr^b^*’ males that have the X-linked *Eif2s3y* transgene and a Y-chromosome with *Sxr^b^* attached distal to the PAR; this cross produces GFP-negative X*^E^Sxr^b^*O and X*^E^*Y*^X^ *Sxr^b^* males with *Sxr^b^* attached to the X PAR. (iii) ‘XY*Sxr^a^*’ males that have a Y-chromosome with *Sxr^a^* attached distal to the PAR; this cross produces GFP-negative X*Sxr^a^*O and XY*^X^ *Sxr^a^* males with *Sxr^a^* attached to the X PAR.
  MF1 XY^RIII^ males were used as normal controls; Y^RIII^ is the strain of Y chromosome from which *Sxr^b^* and *Sxr^a^* derive.
*Zfy1* or *Zfy2* transgenes inserted on autosomes are expressed during pachytene, which results in pachytene stage IV apoptosis and consequent sterility [26], so we have used X-located single copy *Zfy1* and *Zfy2* transgenes that are silenced along with the endogenous X and Y genes at the beginning of pachytene. The *Zfy* and *Zfx* transgene additions involved cross 2(i) except that the X*^E^*Y^Δ*Sry*^ *Sry* male also carried either (i) 1 copy of an X-located *Zfy1*- *Uba1y* BAC (RP24-327G6) transgene [26], (ii) 1 copy of a *Zfy2* BAC inserted by cassette mediated exchange (CME) into the *Hprt* locus on the X chromosome [11], [26], or (iii) 7 copies of an autosomally located *Zfx* BAC (RP23-269L6) transgene.

### Genotyping and copy number estimation

Crosses 1(i)-2(iii) above generate XO males as well as the XY*^X^ males with varying Yp complements. As a guide to the presence of Y*^X^ we utilised PCRs for X-linked *Prdx4* (absent in Y*^X^), *Amelx* (present in Y*^X^) and *Myog* (on chromosome 1) for normalisation. Two PCR reactions were used to detect the presence of Y*^X^ and the number of X-chromosomes. An 82-bp *Prdx4* and a 162-bp *Amelx* fragment were amplified using primers *Prdx4-*F and *Prdx4*-R together with primers *Amelx*-F and *Amelx*-R. The 162-bp *Amelx* fragment and a 246-bp *Myog* fragment were amplified using *Amelx* primers together with *Myog* primers *Om1a* and *Om1b* [72]. Primer sequences are described in Table S4. The following conditions were used: 95°C for 5 min, followed by 28 cycles of 95°C for 30 sec, 60°C for 20 sec and 72°C for 30 sec, with a final extension at 72°C for 5 min. Products were separated on a 3.5% (w/v) agarose gel and the genotype inferred from the relative intensities of the PCR products: XO 1 *Prdx4* + 1 *Amelx* + 2 *Myog*, XY*^X^ 1 *Prdx4* + 2 *Amelx* + 2 *Myog*, XX 2 *Prdx4* + 2 *Amelx* + 2 *Myog* and XXY*^X^ 2 *Prdx4* + 3 *Amelx* + 2 *Myog*.

For mice typed as Y*^X^ positive that provided material for the present study the presence of the Y*^X^ was confirmed either by examination of Giemsa-stained bone marrow chromosome spreads to check for the presence of the very small Y*^X^ chromosome, by SYCP3 and CENT immunostaining of testis cell spreads (see below), or by quantitative PCR using *Prdx4*-F, *Prdx4*-R, *Amelx*-F and *Amelx*-R primers with the following genotypes as controls: XX, XO and XY*^X^. *Om1a* and *Om1b* primers were used for normalisation.

Copy number estimation was done by quantitative PCR as previously described in Royo et al 2010 with slight modification. A SacBII amplicon obtained using SacBII-F and SacBII-R primers (Table S4), match the backbone of the *Zfx*-bearing vector. A X*^Zfy1/Uba1y^*Y sample was used as a reference, because it bears a known transgene copy number of one (*Zfy1-7*; [26]) and the backbone of the *Zfy1/Uba1y*-bearing vector contains SacBII ORF. Reactions were normalised against amplification of the *Atr* gene. The difference in PCR cycles with respect to *Atr* (ΔCt) for a given experimental sample was subtracted from the mean ΔCt of the reference samples (X*^Zfy1/Uba1y^*Y) (ΔΔCt). The transgene copy number was calculated as the mean of the power 2 (ΔΔCt).

### Pairing efficiency

Pairing efficiency between X and Y*^X^ was assessed on surface-spread spermatogenic cells preparation from 6-week-old testes. Briefly, a portion of frozen testicular tissue (approximately 10 mg) was defrosted and macerated in 0.2 ml RPMI 1640 solution (Invitrogen Corporation, Gibco) to produce a thin cell suspension. One drop of cell suspension was applied on a pre-boiled microscope slide, mixed with five drops of 4.5% sucrose solution and allowed to stand for one hour in a humid chamber at room temperature. The cells were permeabilized by adding three drops of 0.05% Triton X-100 solution for 10 min, after which ten drops of 2% formaldehyde solution (TAAB) containing 0.02% SDS pH 8.4 were added for 30 min. The slides were then dipped briefly in distilled water and air-dried. After rehydration in PBS the slides were soaked in PBST-BSA (PBS containing 0.1% Tween 20 and 0.15% BSA) for 1 hour and incubated overnight at 37°C with rabbit polyclonal anti-SYCP3 (1∶300; Abcam) and an anti-centromere (CREST) antibody (1∶500; Antibodies Inc.) diluted in PBST-BSA. Slides were washed in PBST, incubated with chicken anti-rabbit Alexa 488 (1∶500; Molecular Probes) and goat anti-human Alexa 594 (1∶500; Molecular Probes) diluted in PBS for 1 h at 37°C and washed in PBST. Pairing efficiency was evaluated on a Leica microscope after staining the cell nuclei with 4′,6-diamidino-2-phenylindole (DAPI) diluted in the mounting medium (Vectashield with DAPI; Vector).

At least four mice per genotype were used and pairing efficiency was assessed for ∼50 pachytene spermatocytes that were identified based on their DAPI nuclear morphology and their full autosomal synapsis identified by the synaptonemal complex (SYCP3) staining pattern. We classified the pairing of the Y*^X^ in three categories: (i) clear PAR-PAR pairing of the X-chromosome with the Y*^X^ chromosome, (ii) the Y*^X^ chromosome clearly identifiable as a univalent chromosome, and (iii) no Y*^X^ chromosome could be identified (most likely lost during cell spreading).

### Ploidy analysis on testis cell spreads

Nuclear DNA content was measured on surface-spread spermatogenic cells from 6 week old testes as described previously [11], [14] using SYCP3 staining and DAPI fluorescence intensity measurements. Antibody against γH2AFX (1∶500; Upstate) was used to identify the sex body of pachytene spermatocytes [73].

### Fluorescence in situ hybridization (FISH)

RNA-FISH for nascent nuclear transcripts from *Zfy1*, *Zfy2* and *Zfx* was performed as previously described [7], [74] using spread testis cells from adult MF1 male mice. *Zfx* RNA FISH was also carried out on spread testis cells from XY,*Zfx* transgenics. The *Zfy2*-specific probe was BAC CITB-288D7 (Research Genetics), the *Zfy1*-specific probe was a modified version of BAC RP24-498K8 (CHORI) from which we had removed the entire *Uba1y* gene by recombineering, the *Zfx-*specific probe was BAC BMQ-372M23 (CHORI), the *Mtm-*specific probe was BAC RP24-287E17 (CHORI) and the *Uty-*specific probe was BAC CITB-246A22 (Research Genetics). *Zfy1*, *Zfy2* and *Zfx* RNA FISH signals were confirmed with DNA FISH as described previously [74]. Antibody against SYCP3 (1∶100; Abcam) was used to identify secondary spermatocytes as previously describe [11].

### *In vitro* transactivation assay

ZF TA domain-Gal4 fusion-protein constructs were made by inserting cDNA segments encoding the different acidic domains into the *Nco*I and *Sal*I, or *Nde*I and *Sal*I, restriction sites of the vector pGBK-CEN6, a single-copy version of pGBKT7 (Clontech), downstream of the Gal4 DNA-binding domain and the c-myc epitope tag of the vector. pGBK-CEN6 without an insert was included as a negative control. We used a low-copy origin because we had previously noticed that the expression of an acidic domain that strongly transactivates (*Zfy2* and *Gal4*) inhibits yeast growth [7], and this has been described for the overexpression of Gal4 [75]. Validating our strategy, yeast transformed with the different acidic domain constructs, including *Zfy2*, all showed similar growth rates (as did the Gal4 acidic domain – data not shown). To create pGBK-CEN6, we replaced the 2 µ high-copy origin of pGBKT7 with the ARS4/CEN6 low-copy origin from pDEST22 (Invitrogen), by recombineering in the *E.coli* strain DY380 [76].

Acidic domains from human *ZFY* and mouse *Zfy1* and *Zfy2* were transferred to pGBK-CEN6 from pGBKT7 constructs as described previously [7]. The acidic domains from human *ZFX* and mouse *Zfx* were amplified from testis cDNAs and mouse *Zfa* was amplified from genomic DNA. PCR-amplified inserts were shown to be without error by sequencing recombinants. Primers used were *Zfx*: o4472/o4109, with respectively *Nco*I and *Sal*I adaptors, and *ZFX*: o4473/o4109 and *Zfa*: o4471/o4109, with respectively *Nde*I and *Sal*I adaptors (Table S4). One recombinant was selected for each construct and transformed into the *S. cerevisiae* strain Y187, in which the β-galactosidase gene is under the control of the Gal4-responsive Gal1 promoter. Three single transformed colonies were picked from SD/-trp agar plates and grown separately in liquid culture to an OD_600_ of 0.9–1.26 in SD/-trp liquid minimal medium. The β-galactosidase assay was performed on 1 OD_600_ unit of the culture using the permeabilized cell assay [77].

### Statistical analysis

For ploidy frequency the differences between genotypes were assessed by one tail student t-test assuming unequal variances after angular transformation of percentages, using Excel (Microsoft) software. For the transactivation assay one tail student t-test assuming unequal variances was performed on the β-Galactosidase activity.

## Supporting Information

Figure S1 Distribution of *Prm1*, *H2al2y* and *H2al1* transcripts in testis of 2-month old wild type mouse. *In situ* hybridisation using antisense probes for *Prm1*, *H2al2y* or *H2al1* on serial sections of a testis (see Text S1 for experimental procedures). Bottom panel indicates epithelium stages of the corresponding seminiferous tubules (identified using Lectin PNA antibody detection and DAPI staining that are not represented). On the right side of each bright field picture is reported a diagrammatic representation of the expression patterns of each gene with the specified colour code indicative of the relative signal intensity of the probe; ranging from very faint, faint, moderate to strong expression. The scale bar represents 160 µm.
(TIF)

Figure S2 Transcription of the autosomally-located *Zfx* transgene assessed by RNA FISH for nascent nuclear transcripts. Representative images of pachytene spermatocyte (Pa) and secondary spermatocyte (SS) nuclei from a 6-week old male bearing an autosomally-located *Zfx* transgene are shown hybridized with RNA FISH probes specific for *Zfx* (arrows) and stained with an antibody against SYCP3 as indicated. X- or Y-bearing cells were differentiated using X-paint labelling. A. A pachytene spermatocyte expressing the *Zfx* transgene next to a *Zfx* expressing Sertoli cell (Sert). B. To confirm autosomally-located *Zfx* transgene expression in pachytene spermatocytes, staining of sex body with γH2AFX antibody was also used. C. Representative images of a Y-bearing secondary spermatocyte (no X-paint labelling) expressing the *Zfx* transgene. D. Numbers of X- or Y-bearing secondary spermatocytes scored as positive or negative for *Zfx* expression. † 4 out of 17 secondary spermatocytes were expressing only the autosomally-located *Zfx* transgene (see Text S1 for detailed experimental procedures).
(TIF)

Figure S3 Western blot analysis of proteins extracted from yeast cells transformed with the seven constructs used to assess the transactivation activity of ZF protein acidic domains. Western blot analysis with anti c-myc antibody (Text S1) shows the presence of the ZF fusion proteins from the six different ZF isoforms from humans (hs) or mouse (mm). Series1–3 are the three transformed colonies used for each construct. The few observed differences in fusion protein concentration between transformants carrying the same construct did not correlate with β-galactosidase activity (Figure 6). However, the mm ZFX and mm ZFY1 fusion protein concentrations were higher than that of mm ZFY2 in the three series. We conclude from this that the fusion protein concentration is probably not limiting for the transactivation in any transformant and that it would therefore be inappropriate to normalise β-galactosidase activity to fusion gene concentration. mm ZFA is encoded by an autosomal gene derived from a retroposed X transcript. Molecular weights (MW) based on the size standard are shown in the first lane. The expected sizes of fusion proteins range from about 52 kDa for mm ZFA to 60 kDa for hs ZFX, hs ZFY and mm ZFX. The retarded migration of the ZF fusion proteins is most likely a consequence of the large positively charged acidic domain [7].
(TIF)

Figure S4 *Zfx* transcription in mid/late zygotene spermatocytes. The two pictures are of the same mid/late zygotene spermatocyte nucleus, showing the robust RNA FISH signal (green) obtained with the *Zfx* probe. The staining for phospho-H2AFX (left) followed by staining for SYCP3 (right) enables a confident assessment of meiotic stage. All 25 mid/late zygotene cells analyzed had robust *Zfx* RNA FISH signals.
(TIF)

Figure S5 Markedly increased MI apoptosis in 30 day old X*^E^*OSry male transgenic for *Zfx*. A. TUNEL-positive (green) first-meiotic metaphases (MIs) and healthy phospho histone H3 (pH3, red) positive MIs were identified by their position away from the basal cell layers. As previously shown [11] there are relatively few apoptotic cells in X*^E^*O*Sry* testes, and these are predominantly spermatogonia located at the periphery of the tubules. With the addition of the *Zfx* transgene there are now abundant more centrally-located apoptotic cells, which are apoptotic MI spermatocytes in stage XII tubules. DAPI (blue) was used as a nuclear stain (see Text S1 for detailed experimental procedures). The scale bar represents 200 µm. B. Quantitation of MI apoptosis was carried out on entire testis sections (16 to 46 seminiferous tubules with MI) from X*^E^*O*Sry* and X*^E^*O*Sry*,*Zfx* mice as previously described [11]. *Zfx* transgene addition is effective in promoting the apoptotic response at MI when added to X*^E^*O*Sry* males. *p≤0.05, **p≤0.01 and ***p≤0.001.
(TIF)

Table S1 Strategy for adjusting the haploid frequencies of the Y*^X^-bearing males to remove the products of the MI cells that did not achieve PAR-PAR synapsis.
(XLS)

Table S2 Haploid spermatid frequencies in XY mice, and in XO and XY*^X^ mice with varying Yp gene complements.
(DOC)

Table S3 X- and Y-linked gene expression by RNA-FISH in spermatogenic cells from adult XY male.
(DOC)

Table S4 List of primers used to amplify the acidic domains from human and mouse ZF proteins.
(DOC)

Text S1 Supplemental experimental procedures.
(DOCX)
