# Supplementary figures and images for: Mouse Y-Linked Zfy1 and Zfy2 Are Expressed during the Male-Specific Interphase between Meiosis I and Meiosis II and Promote the 2nd Meiotic Division
Source: PLoS Genet. 2014 Jun 26;10(6):e1004444. doi: 10.1371/journal.pgen.1004444 (PMC4072562; doi:10.1371/journal.pgen.1004444)

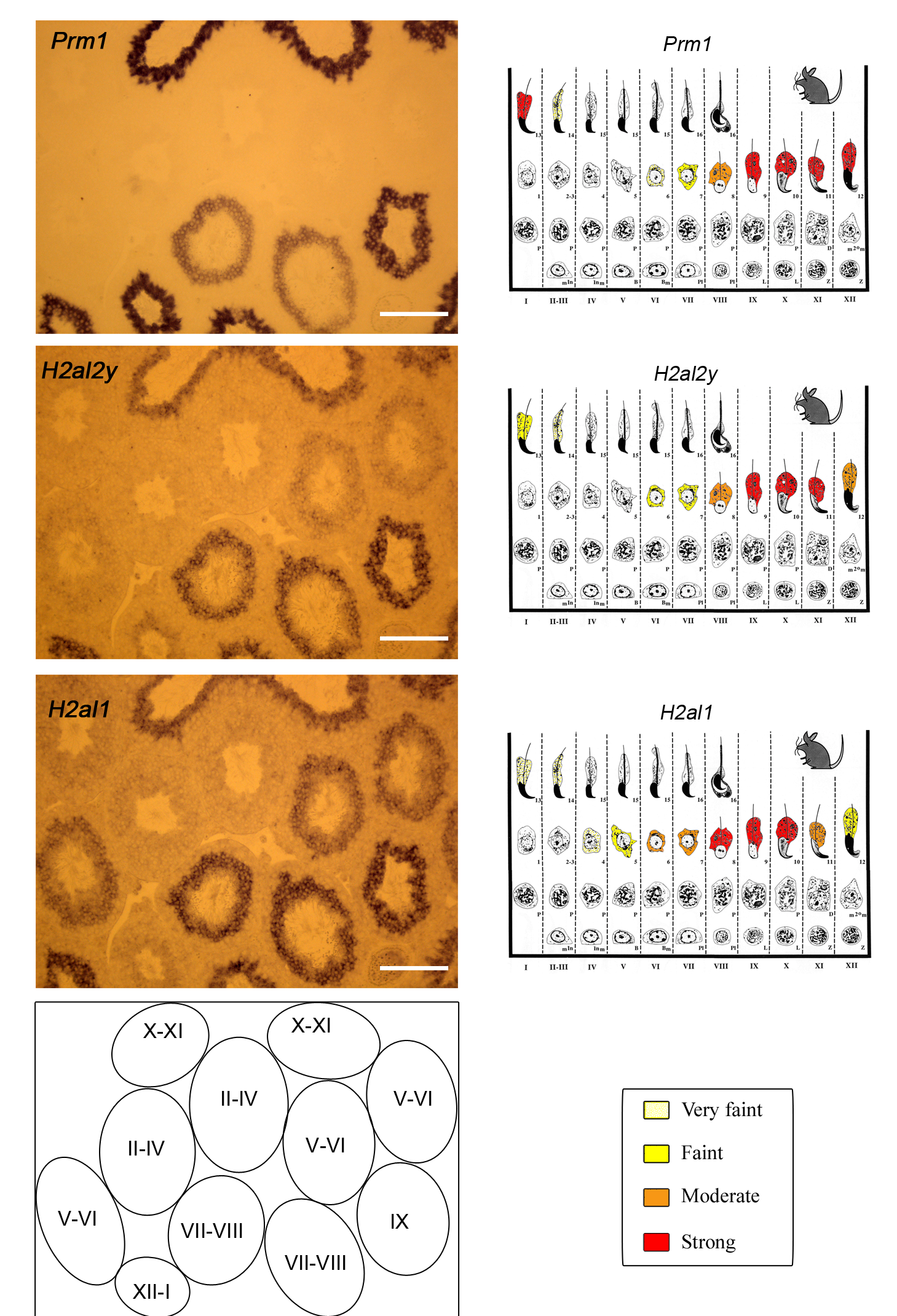

Supplement: Figure S1 — Distribution of Prm1, H2al2y and H2al1 transcripts in testis of 2-month old wild type mouse. In situ hybridisation using antisense probes for Prm1, H2al2y or H2al1 on serial sections of a testis (see Text S1 for experimental procedures). Bottom panel indicates epithelium stages of the corresponding seminiferous tubules (identified using Lectin PNA antibody detection and DAPI staining that are not represented). On the right side of each bright field picture is reported a diagrammatic representation of the expression patterns of each gene with the specified colour code indicative of the relative signal intensity of the probe; ranging from very faint, faint, moderate to strong expression. The scale bar represents 160 µm. (TIF) [file pgen.1004444.s001.tif]

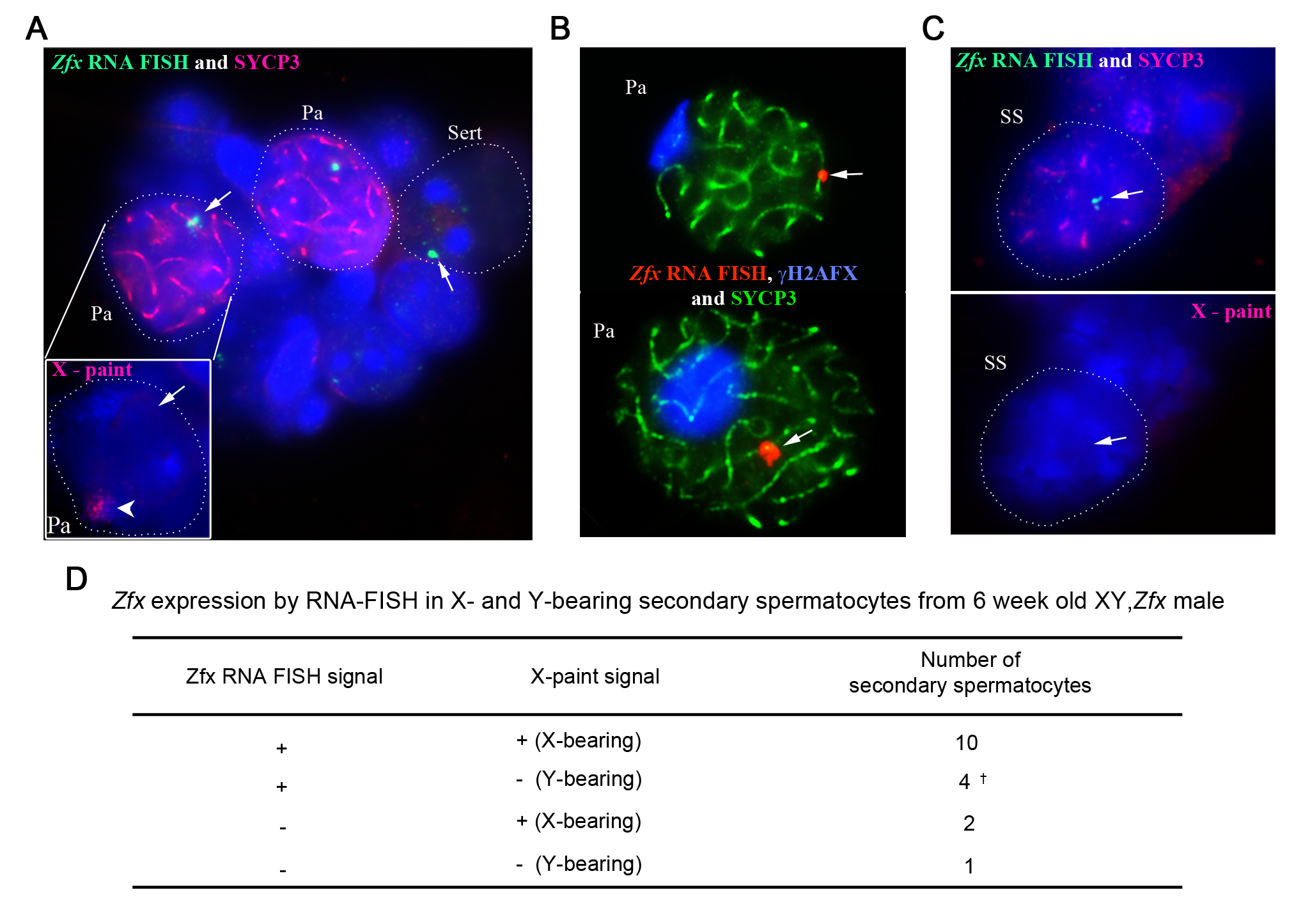

Supplement: Figure S2 — Transcription of the autosomally-located Zfx transgene assessed by RNA FISH for nascent nuclear transcripts. Representative images of pachytene spermatocyte (Pa) and secondary spermatocyte (SS) nuclei from a 6-week old male bearing an autosomally-located Zfx transgene are shown hybridized with RNA FISH probes specific for Zfx (arrows) and stained with an antibody against SYCP3 as indicated. X- or Y-bearing cells were differentiated using X-paint labelling. A. A pachytene spermatocyte expressing the Zfx transgene next to a Zfx expressing Sertoli cell (Sert). B. To confirm autosomally-located Zfx transgene expression in pachytene spermatocytes, staining of sex body with γH2AFX antibody was also used. C. Representative images of a Y-bearing secondary spermatocyte (no X-paint labelling) expressing the Zfx transgene. D. Numbers of X- or Y-bearing secondary spermatocytes scored as positive or negative for Zfx expression. † 4 out of 17 secondary spermatocytes were expressing only the autosomally-located Zfx transgene (see Text S1 for detailed experimental procedures). (TIF) [file pgen.1004444.s002.tif]

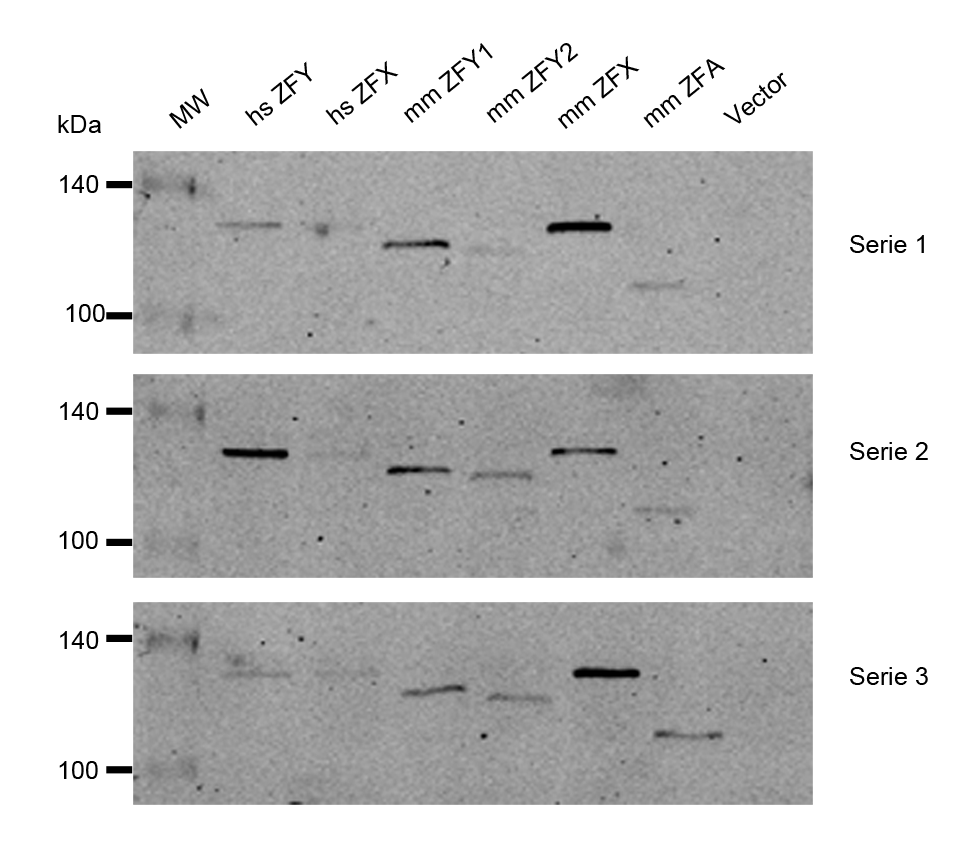

Supplement: Figure S3 — Western blot analysis of proteins extracted from yeast cells transformed with the seven constructs used to assess the transactivation activity of ZF protein acidic domains. Western blot analysis with anti c-myc antibody (Text S1) shows the presence of the ZF fusion proteins from the six different ZF isoforms from humans (hs) or mouse (mm). Series1–3 are the three transformed colonies used for each construct. The few observed differences in fusion protein concentration between transformants carrying the same construct did not correlate with β-galactosidase activity (Figure 6). However, the mm ZFX and mm ZFY1 fusion protein concentrations were higher than that of mm ZFY2 in the three series. We conclude from this that the fusion protein concentration is probably not limiting for the transactivation in any transformant and that it would therefore be inappropriate to normalise β-galactosidase activity to fusion gene concentration. mm ZFA is encoded by an autosomal gene derived from a retroposed X transcript. Molecular weights (MW) based on the size standard are shown in the first lane. The expected sizes of fusion proteins range from about 52 kDa for mm ZFA to 60 kDa for hs ZFX, hs ZFY and mm ZFX. The retarded migration of the ZF fusion proteins is most likely a consequence of the large positively charged acidic domain [7]. (TIF) [file pgen.1004444.s003.tif]

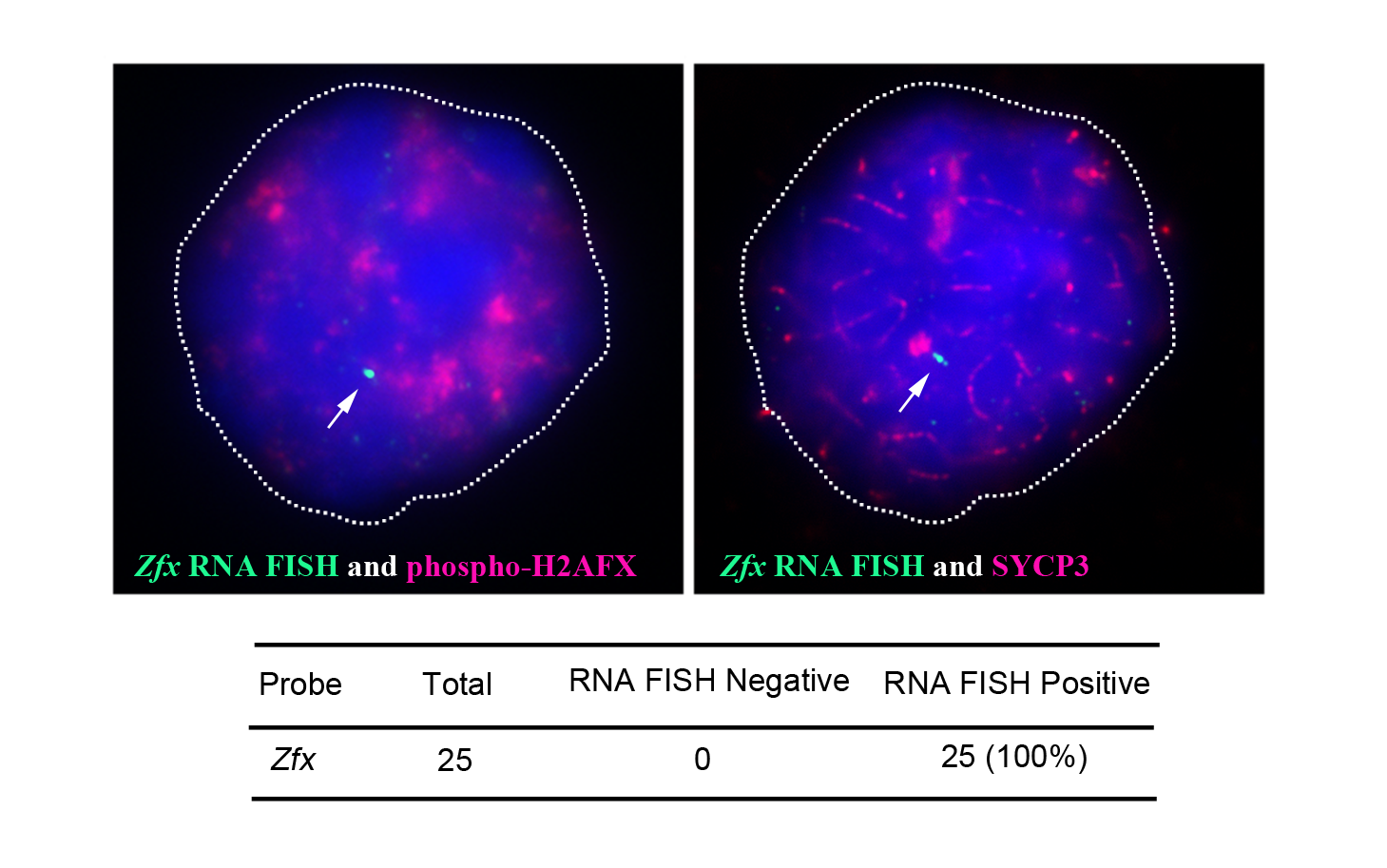

Supplement: Figure S4 — Zfx transcription in mid/late zygotene spermatocytes. The two pictures are of the same mid/late zygotene spermatocyte nucleus, showing the robust RNA FISH signal (green) obtained with the Zfx probe. The staining for phospho-H2AFX (left) followed by staining for SYCP3 (right) enables a confident assessment of meiotic stage. All 25 mid/late zygotene cells analyzed had robust Zfx RNA FISH signals. (TIF) [file pgen.1004444.s004.tif]

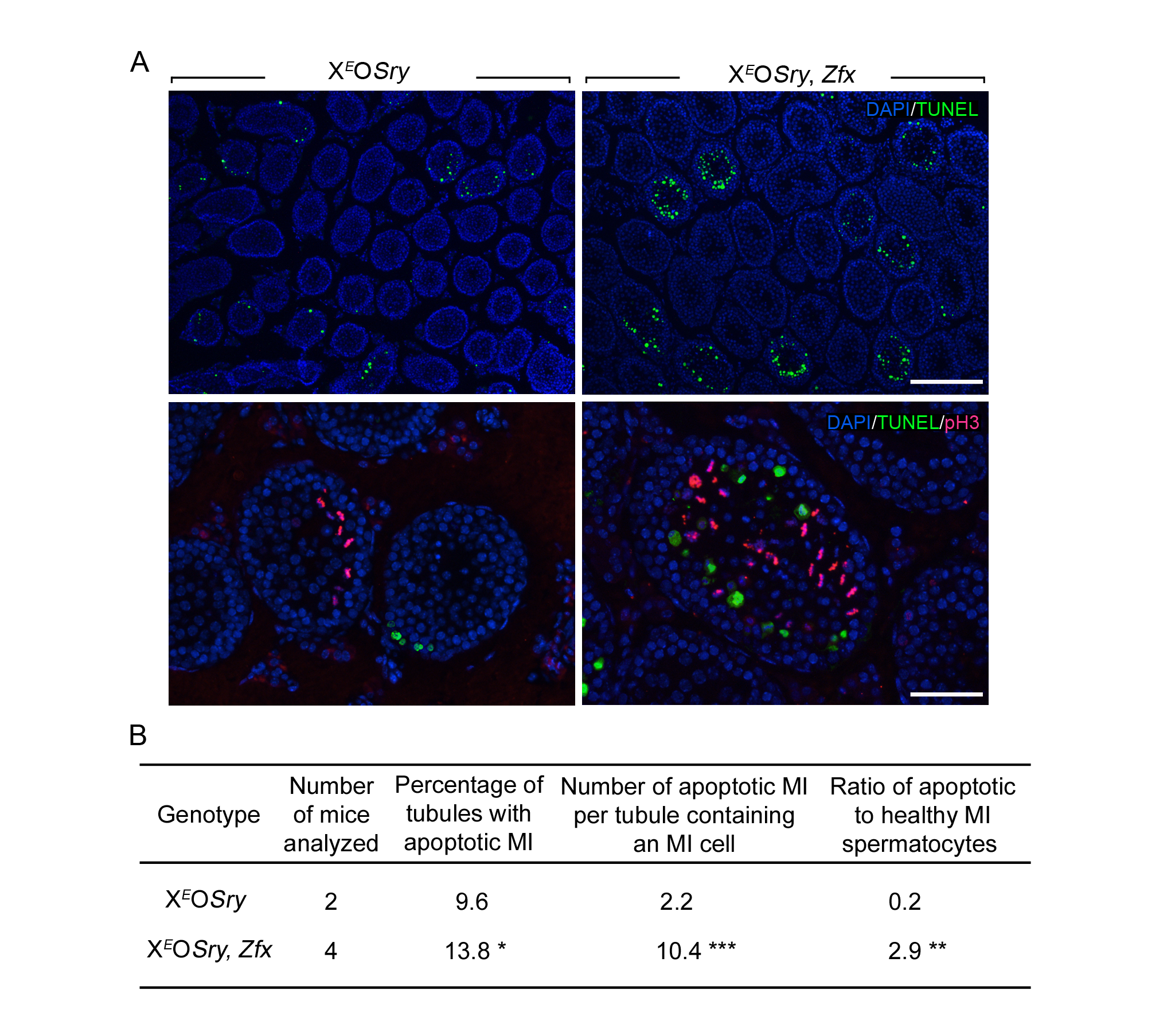

Supplement: Figure S5 — Markedly increased MI apoptosis in 30 day old XEOSry male transgenic for Zfx. A. TUNEL-positive (green) first-meiotic metaphases (MIs) and healthy phospho histone H3 (pH3, red) positive MIs were identified by their position away from the basal cell layers. As previously shown [11] there are relatively few apoptotic cells in XEOSry testes, and these are predominantly spermatogonia located at the periphery of the tubules. With the addition of the Zfx transgene there are now abundant more centrally-located apoptotic cells, which are apoptotic MI spermatocytes in stage XII tubules. DAPI (blue) was used as a nuclear stain (see Text S1 for detailed experimental procedures). The scale bar represents 200 µm. B. Quantitation of MI apoptosis was carried out on entire testis sections (16 to 46 seminiferous tubules with MI) from XEOSry and XEOSry,Zfx mice as previously described [11]. Zfx transgene addition is effective in promoting the apoptotic response at MI when added to XEOSry males. *p≤0.05, **p≤0.01 and ***p≤0.001. (TIF) [file pgen.1004444.s005.tif]
