# Supplementary material for: Mouse Y-Linked Zfy1 and Zfy2 Are Expressed during the Male-Specific Interphase between Meiosis I and Meiosis II and Promote the 2nd Meiotic Division
Source: PLoS Genet. 2014 Jun 26;10(6):e1004444. doi: 10.1371/journal.pgen.1004444 (PMC4072562; doi:10.1371/journal.pgen.1004444)
Supplement: Table S2 — Haploid spermatid frequencies in XY mice, and in XO and XY*X mice with varying Yp gene complements. (DOC) [file pgen.1004444.s007.doc]

**Suppl Table 2.** Haploid spermatid frequencies in XY mice, and in XO and XY*^X^ mice with varying Yp gene complements.

A

| Males used in [14] | |
| --- | --- |
| Genotype ^a^ | % Haploid ^a^ |
| **X*Sxr^a^*O** | Pooled data: **44.1%** |
| 1 | 21.3% (n=61) |
| 2 | 47.9% (n=73) |
| 3 | 50.5% (n=97) |
| 4 | 52.6% (n=57) |
| **X*^E^Sxr^b^*O** | Pooled data: **5.2%** |
| 1 | 1.5% (n=66) |
| 2 | 5.7% (n=70) |
| 3 | 6.3% (n=64) |
| 4 | 6.9% (n=87) |
| **X*^E^*O*Sry*** | Pooled data: **11.4%** |
| 1 | 5.4% (n=56) |
| 2 | 6.9% (n=58) |
| 3 | 14.1% (n=92) |
| 4 | 18.4% (n=49) |
| **XY** | Pooled data: **99.1%** |
| 1 | 97.5% (n=80) |
| 2 | 100.0% (n=70) |
| 3 | 100.0% (n=74) |

B

| Males used in [14] + Y*^X^ | |
| --- | --- |
| **Genotype** | % Haploid |
| **XY*^X^*Sxr^a^*** | Pooled data: **96.0%** |
| 1 | 93.0% (n=158) |
| 2 | 96.1% (n=102) |
| 3 | 97.2% (n=142) |
| 4 | 98.9% (n=94) |
| **X*^E^*Y*^X^*Sxr^b^*** | Pooled data: **44.5%** |
| 1 | 28.6% (n=126) |
| 2 | 45.1% (n=193) |
| 3 | 49.0% (n=143) |
| 4 | 56.6% (n=106) |
| **X*^E^*Y*^X^*Sry*** | Pooled data: **15.9%** |
| 1 | 7.4% (n=68) |
| 2 | 11.6% (n=86) |
| 3 | 17.5% (n=57) |
| 4 | 23.0% (n=122) |

C

| X*^E^*Y*^X^*Sry* males + *Zfy* transgene | |
| --- | --- |
| Genotype | % Haploid |
| **X*^E^*Y*^X^*Sry*,*Zfy1*** | Pooled data: **39.6%** |
| 1 | 28.3% (n=258) |
| 2 | 28.4% (n=109) |
| 3 | 35.0% (n=100) |
| 4 | 58.1% (n=246) |
| **X*^E^*Y*^X^*Sry*,*Zfy2*** | Pooled data: **78.2%** |
| 1 | 75.2% (n=298) |
| 2 | 76.9% (n=121) |
| 3 | 81.9% (n=116) |
| 4 | 88.5% (n=61) |
| **X*^E^*Y*^X^*Sry*,*Zfy1*,*Zfy2*** | Pooled data: **87.6%** |
| 1 | 69.2% (n=182) |
| 2 | 94.4% (n=54) |
| 3 | 94.8% (n=172) |
| 4 | 96.8% (n=189) |

D

| X*^E^*Y*^X^*Sry* males +/- *Zfx* transgene | |
| --- | --- |
| Genotype | % Haploid |
| **X*^E^*Y*^X^*Sry*** | Pooled data: **14.1%** |
| 1 | 8.4% (n=155) |
| 2 | 13.6% (n=132) |
| 3 | 16.1% (n=143) |
| 4 | 18.4% (n=158) |
| **X*^E^*Y*^X^*Sry*,*Zfx*** | Pooled data: **79.9%** |
| 1 | 73.6% (n=231) |
| 2 | 77.6% (n=147) |
| 3 | 83.0% (n=153) |
| 4 | 86.6% (n=194) |

The data were collected after DNA quantitation using DAPI fluorescence intensity measurement on SYCP3-labelled testis cell spreads.

Genotype averages are calculated as the percentages of pooled data.

^a^ Data already published in [14]
