# Supplementary material for: Mouse Y-Linked Zfy1 and Zfy2 Are Expressed during the Male-Specific Interphase between Meiosis I and Meiosis II and Promote the 2nd Meiotic Division
Source: PLoS Genet. 2014 Jun 26;10(6):e1004444. doi: 10.1371/journal.pgen.1004444 (PMC4072562; doi:10.1371/journal.pgen.1004444)
Supplement: Table S3 — X- and Y-linked gene expression by RNA-FISH in spermatogenic cells from adult XY male. (DOC) [file pgen.1004444.s008.doc]

**Table S3.** **X- and Y-linked gene expression by RNA-FISH in spermatogenic cells from adult XY male.**

| Probe | Interphasic secondary spermatocytes | | | Spermatids | | |
| --- | --- | --- | --- | --- | --- | --- |
|  | Total | RNA FISH Negative | RNA FISH Positive ^a^  (% of positive ^b^) | Total | RNA FISH Negative | RNA FISH Positive ^a^  (% of positive ^b^) |
| *Uty* | 25 | 14 | 11 (88%) | 30 | 21 | 9 (60%) |
| *Mtm1* | 24 | 16 | 8 (67%) | 33 | 27 | 6 (36%) |
| *Zfx* | 31 | 17 | 14 (90%) | 31 | 26 | 5 (32%) |

^a^ Data include X and Y-carrying secondary spermatocytes and spermatids because the cells have not been scored with X or Y DNA FISH.

^b^ Percentages of cells expressing have been calculated assuming that one half of the cells scored were X-carrying.
