# Supplementary material for: Mouse Y-Linked Zfy1 and Zfy2 Are Expressed during the Male-Specific Interphase between Meiosis I and Meiosis II and Promote the 2nd Meiotic Division
Source: PLoS Genet. 2014 Jun 26;10(6):e1004444. doi: 10.1371/journal.pgen.1004444 (PMC4072562; doi:10.1371/journal.pgen.1004444)
Supplement: Table S4 — List of primers used to amplify the acidic domains from human and mouse ZF proteins. (DOC) [file pgen.1004444.s009.doc]

**Table S4.** **List of primers used to amplify the acidic domains from human and mouse ZF proteins.**

| Gene | Primer ID | Primer sequence | Amplicon  size |  |
| --- | --- | --- | --- | --- |
| **Primers for genotyping and copy number estimation** | | | | |
| *Prdx4* | *Prdx4*‑F | CATGATATCCACTGAAAGCTAC | 82bp |  |
|  | *Prdx4*‑R | GAGACAGTGTATCTATCCCTG |  |  |
| *Amelx* | *Amelx*‑F | GTTGGGTTGGAGTCATGGAG | 162bp |  |
|  | *Amelx*‑R | GGCTGCACCACCAAATCATC |  |  |
| *Myog* | *Om1a* | TTACGTCCATCGTGGACAGCAT | 246bp |  |
|  | *Om1b* | TGGGCTGGGTGTTAGCCTTAT |  |  |
| SacBII | SacBII‑F | GCTGCAGATCCCTAAACAGC | 515bp |  |
|  | SacBII‑R | TTCCGTCTCCGTCAAAAATC |  |  |
| **Primers for cloning Zf acidic domains** | | | | |
| *ZFX* | o4473-*NdeI* adaptor | CCTTGA*CATATG*GATGAAGATGGGCTTG | 1195bp |  |
|  | o4109-*Sal*I adaptor | AAGGTC*GTCGAC*TGCCAAGGCCAGCAGACTCATCTATGTG |  |  |
| *Zfx* | o4472-*Nco*I adaptor | TAAAGG*CCATGG*ATGAAGATGG | 1176bp |  |
|  | o4109-*Sal*I adaptor | AAGGTC*GTCGAC*TGCCAAGGCCAGCAGACTCATCTATGTG |  |  |
| *Zfa* | o4471-*NdeI* adaptor | GCTCCA*CATATG*GATGGTAGTC | 934bp |  |
|  | o4109-*Sal*I adaptor | AAGGTC*GTCGAC*TGCCAAGGCCAGCAGACTCATCTATGTG |  |  |
