# Supplementary material for: Mouse Y-Linked Zfy1 and Zfy2 Are Expressed during the Male-Specific Interphase between Meiosis I and Meiosis II and Promote the 2nd Meiotic Division
Source: PLoS Genet. 2014 Jun 26;10(6):e1004444. doi: 10.1371/journal.pgen.1004444 (PMC4072562; doi:10.1371/journal.pgen.1004444)
Supplement: Text S1 — Supplemental experimental procedures. (DOCX) [file pgen.1004444.s010.docx]

**[Text S1.](http://www.plosgenetics.org/article/fetchSingleRepresentation.action?uri=info:doi/10.1371/journal.pgen.1000421.s007)** **Supplemental experimental procedures**

**X chromosome and sex body detection after RNA‑FISH**

When studying the autosomal *Zfx* transgene expression in secondary spermatocytes, mouse IDetect^TM^ X chromosome paint (ID Labs Inc) was used to identify X-bearing secondary spermatocytes. Briefly, the slides used for the *Zfx* transgene FISH analysis were rinsed in 1x PBS, de‑hydrated in ethanol (70‑100% series) and air-dried. 15µl of X chromosome paint was applied to each slide, denatured at 70^o^C for 2 minutes, and incubated overnight at 37^o^C. Slides were then washed at 45^o^C with four 3 minute washes in 2xSSC, followed by four 3 minute washes in 0.01xSSC. Slides were mounted in DAPI for subsequent analysis. Antibody against γH2AFX (1/500; Upstate) was used to identify the sex body of pachytene spermatocytes [73].

**RNA in situ hybridisation**

RNA in situ hybridisation with digoxigenin-labeled probes for transcripts from *Prm1*, *H2al2y* (2Y copies) and *H2al1* (1X copy) was performed as described in [78] with slight modifications. Briefly, testes were fixed in 4% PFA overnight at 4°C and rinsed with 70% ethanol before embedding in paraffin. Dewaxed and rehydrated sections were incubated overnight at 65°C in a humidified chamber with 100μl of hybridization solution [50% formamide, 10% dextran sulphate, 1X Denhardt’s solution, 200 mM sodium chloride, 5 mM EDTA pH 8.0, 10 mM tris buffer pH 7.5, 11.5 mM sodium phosphate buffer pH 6.4 and tRNA 1 mg/ml] containing the digoxigenin labelled riboprobe. Posthybridization washes and staining were done as described in [78]. Nuclei were counterstained with DAPI in a mounting medium (Vectashield with DAPI; Vector). For riboprobe synthesis, parts of the coding region were amplified from testis cDNA using forward primer *Prm1*‑F (5’‑GAC ACA GCC CAC AAA ATT CC‑3’) and reverse primer *Prm1*‑R (5’‑GAC AGG TGG CAT TGT TCC TT‑3’) to amplify *Prm1* (385bp) and forward primer *H2al2y*‑F (5’‑AGA TGG AAA ATA ACC CAC TCC‑3’) and reverse primer *H2al2y*‑R (5’‑CAA ATT CCC CAC ATT AAC GA‑3’) to amplify *H2al2y* (533bp). PCR fragments were cloned into pCR II TOPO vector using TOPO TA cloning kit (Invitrogen). Plasmid containing *H2al1* (205bp) was already described in [79] (probe against AK005922). The plasmids containing *Prm1*, *H2al2y* and *H2al1* cDNA were linearized and used as templates for the synthesis of the sense or antisense digoxigenin labelled riboprobes as described [78].

**TUNEL assays**

Apoptotic cells were detected using the terminal deoxynucleotidyl transferase dUTP nick end-labeling (TUNEL) assay (In Situ Cell Death Detection Kit, fluorescein; Roche). Briefly, testis samples were fixed in 4% PFA overnight at 4°C and embedded in paraffin. 5 µm sections on glass slides were de‑waxed and incubated with TUNEL staining solution for 1 hour at room temperature. Nuclei were counterstained with Vectashield containing 4',6‑diamidino-2-phenylindole (DAPI, Vector).

**Protein extraction and Western blot analysis**

Proteins were prepared from 2.5 OD_600_ units of culture of yeast cells transformed with the six constructs used to assess the transactivation capacity of the acidic domains of ZF proteins. Yeast protein extracts were run on a 6% SDS/polyacrylamide gel. After transfer and blocking, membranes were incubated overnight with anti-human c‑myc tag antibody (BioLegend). Incubation with donkey anti-mouse IRDye 800CW fluorescent secondary antibody (LiCor), and signals were detected using the Odyssey Infrared Imaging System (LiCor). Loading was controlled to be equal by staining of the blotted membrane with Ponceau S.

**Additional references**

78. Vernet N, Dennefeld C, Rochette-Egly C, Oulad-Abdelghani M, Chambon P, et al. (2006) Retinoic acid metabolism and signaling pathways in the adult and developing mouse testis. Endocrinology 147: 96-110.

79. Ellis PJI, Clemente EJ, Ball P, Toure A, Ferguson L, et al. (2005) Deletions on mouse Yq lead to upregulation of multiple X- and Y-linked transcripts in spermatids. Human Molecular Genetics 14: 2705-2715
